# Plakinamine P, A Steroidal Alkaloid with Bactericidal Activity against *Mycobacterium tuberculosis*

**DOI:** 10.3390/md17120707

**Published:** 2019-12-16

**Authors:** Carolina Rodrigues Felix, Jill C. Roberts, Priscilla L. Winder, Rashmi Gupta, M. Cristina Diaz, Shirley A. Pomponi, Amy E. Wright, Kyle H. Rohde

**Affiliations:** 1 Biotechnology Department, Universidade Federal de Pelotas, Pelotas, RS 96010-610, Brazil; carolinarodriguesfelix@gmail.com; 2 Harbor Branch Oceanographic Institute, Florida Atlantic University, Fort Pierce, FL 34946, USA; jrober90@fau.edu (J.C.R.); PWINDER@fau.edu (P.L.W.); taxochica@gmail.com (M.C.D.); spomponi@fau.edu (S.A.P.); 3 Division of Immunity and Pathogenesis, Burnett School of Biomedical Sciences, College of Medicine, University of Central Florida, Orlando, FL 32827, USA; rashmi.gupta@ucf.edu

**Keywords:** marine compounds, tuberculosis, drug discovery

## Abstract

Tuberculosis is the leading cause of death due to infectious disease worldwide. There is an urgent need for more effective compounds against this pathogen to control the disease. Investigation of the anti-mycobacterial activity of a deep-water sponge of the genus *Plakina* revealed the presence of a new steroidal alkaloid of the plakinamine class, which we have given the common name plakinamine P. Its structure is most similar to plakinamine L, which also has an acyclic side chain. Careful dissection of the nuclear magnetic resonance data, collected in multiple solvents, suggests that the dimethyl amino group at the 3 position is in an equatorial rather than axial position unlike previously reported plakinamines. Plakinamine P was bactericidal against *M. tuberculosis*, and exhibited moderate activity against other mycobacterial pathogens, such as *M. abscessus* and *M. avium*. Furthermore, it had low toxicity against J774 macrophages, yielding a selectivity index (SI, or IC_50_/MIC) of 8.4. In conclusion, this work provides a promising scaffold to the tuberculosis drug discovery pipeline. Future work to determine the molecular target of this compound may reveal a pathway essential for *M. tuberculosis* survival during infection.

## 1. Introduction

*Mycobacterium tuberculosis* (*Mtb*) is the causative agent of the primarily pulmonary disease tuberculosis (TB). Approximately 10 million new cases of TB are diagnosed every year worldwide, and 5% of these are caused by multidrug-resistant strains of *Mtb*, rendering the disease extremely difficult to control [1]. TB treatment entails a combination of four antibiotics taken for at least six months in order to achieve a relapse-free cure [2]. Considering this, there is an urgent need for novel compounds that are more effective against this pathogen, capable of shortening treatment time, and killing drug-resistant strains of *Mtb*.

Recently, a large-scale screen of a marine natural products (MNP) library containing 4400 pre-fractionated peak fraction samples yielded highly potent inhibitors of *Mtb* [3]. A sample from the marine invertebrate *Plakina* sp. was highly active in the primary screen and was chosen for further deconvolution in this study. A novel plakinamine was discovered with potent bactericidal activity against *Mtb*. We have elucidated the structure of this novel compound and further characterized its activity against *Mtb* as well as other important mycobacterial pathogens, including *M. abscessus*, and two species belonging to the *M. avium* complex. The plakinamine compound was selectively active against *Mtb*, though moderate activity was observed against the other mycobacteria tested. Investigating the mechanism of action of this compound may reveal novel druggable *Mtb* targets. The structural similarity of plakinamine P and cholesterol, coupled with the essential nature of the cholesterol catabolism pathway for *Mtb* survival during infection, poses an interesting pool of putative targets for the novel structure described in this study [4].

## 2. Results

### 2.1. Chemical Analysis

A specimen of sponge identified as a new species of *Plakina* was collected using the Johnson Sea Link I manned submersible at a depth of 93 m off Crooked Island, the Bahamas, and stored at −20 °C until workup. The frozen sponge was extracted using a Dionex Accelerated Solvent Extractor and a series of solvents with increasing polarity. The CH_3_OH:H_2_O (3:1 v/v) extract was further purified using reverse-phase flash chromatography on a Teledyne Isco CombiFlash Rf 4x, followed by semi-preparative HPLC leading to the isolation of plakinamine P (**1**) as a tan oil [α]_D_^20^ = + 16.9 (*c* 0.11 in MeOH).

**Figure 1:**
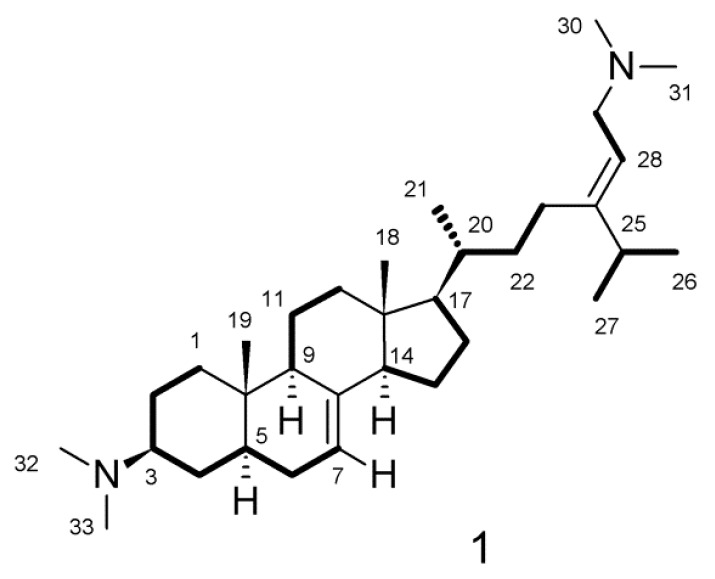
Thick lines indicate spins systems defined through interpretation of the 2D-COSY and edited gHSQC NMR spectra.

Direct analysis in real time–high resolution mass spectrometry (DART-HRMS) analysis of **1** coupled with interpretation of the ^13^C nuclear magnetic resonance (NMR) spectrum suggested a molecular formula of C_33_H_58_N_2_ requiring six degrees of unsaturation. The ^1^H NMR spectra of **1** had substantial overlap and, therefore, data were collected in methanol-*d*_4_ and dimethyl-sulfoxide (DMSO)-*d*_6_ to allow for assignment of all atoms (Supplementary Figures S1–S24, Table S1 and S2). The ^1^H and ^13^C NMR spectra collected in methanol-*d*_4_ showed the presence of four sp^2^ hybridized carbons [*δ*_C_ 159.9, 140.7, 118.4 and 111.7] consistent with two double bonds. No additional unsaturation was apparent from the NMR data, suggesting that **1** has four rings. The ^1^H NMR spectrum showed the presence of an isopropyl group [*δ*_H_ 2.38 sep (*J* = 6.9 Hz), *δ*_H_ 1.10 3H d (*J* = 6.9 Hz), *δ*_H_ 1.09 d 3H (*J* = 6.9 Hz)]. The presence of two dimethylamino groups in **1** was suggested by the resonances observed as two broadened singlets (δ_H_ 2.84 and δ_H_ 2.85) integrating for 12 protons attached to carbon resonances observed at *δ*_C_ 42.7 and 40.5. A literature search suggested that similar resonances are found in the plakinamine class of natural products [5,6,7,8,9,10,11,12,13,14,15] and comparison of the NMR data to the published data suggested that **1** is most similar to plakinamines L and M [14,15]. Other characteristic resonances observed in the ^1^H NMR spectrum of **1** were the methyl resonances observed at δ_H_ 0.59 (H_3_-18, s) and δ_H_ 0.86 (H_3_-19, s), which are consistent with the angular methyl groups of a steroidal alkaloid. Analysis of the 2D-COSY and 2D-edited gHSQC NMR spectra allowed for the assignment of five spin systems shown in Figure 1 as thickened bonds: C-1 through C-7; C-9 → C-11 → C-12; C-14 though C-17 → C-20 through C-23; C-25 → C-26 and C-27; and C-28 → C-29. The fragments were connected through the analysis of the 2D gHMBC spectrum (Figures S9–S12).

Correlations observed in the HMBC spectrum from H-6a to C-8 assign the position of the olefinic carbon C-8. Correlations observed between H-7 and C-9; between H_3_-19 and C-1, C-5, C-9, and C-10, and between H-1b and C-19 defined the presence of fused rings A and B and the position of the C-19 methyl group. Correlations in the HMBC spectrum from H-7 to C-14; H-11ab to C-8; H-12a to C-13 and C-14; H-14 to C-8 and C-13, and from H_3_-18 to C-12, C-13, C-14, and C-17 established rings C and D as well as the position of C-18. The structure of the side chain attached at C-17 was established based on correlations in the HMBC spectrum from H-23ab to C-24, C-25, and C-28; from H-25 to C-23, C-24, and C-28; and from H-28 to C-23, C-24, and C-25. The placement of the two dimethylamino groups was determined based on HMBC correlations from H_3_-30/31 to C-29 in the acyclic side chain and from H_3_-32/33 to C-3 of ring A. Detailed results from NMR analysis are shown in Table 1.

**Figure 2:**
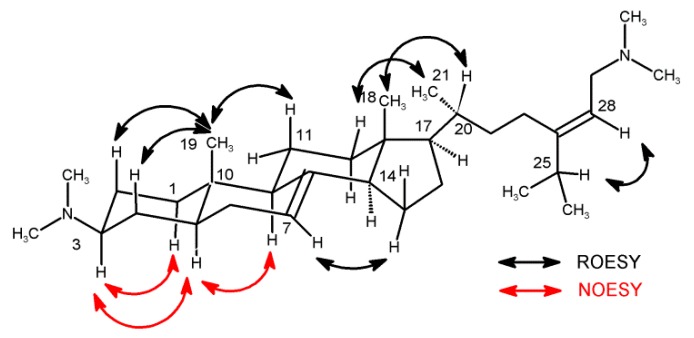
Key ROESY and NOESY correlations for **1** observed in dimethyl-sulfoxide (DMSO)-*d*_6_.

The relative configuration of **1** was defined based upon the ROESY and NOESY data (Figure 2 and Supplementary S13, S21-S23, Table S2) as well as comparison with previously described plakinamines [5,6,7,8,9,10,11,12,13,14,15]. NMR data were collected in two different solvents to fully resolve the A ring protons with DMSO- *d_6_* yielding sufficient resolution. NOESY correlations observed between H-3 (δ_H_ 3.07), H-1b (δ_H_ 1.07), and H-5 (δ_H_ 1.37) were consistent with axial orientations for all three protons. This differs from other reported plakinamines in which the H-3 proton is equatorial. A *trans*-ring-fusion of the A and B rings indicating a chair conformation was established based on NOESY correlations between the axial protons H-5 (δ_H_ 1.37) and H-9 (δ_H_ 1.64) along with 1,3-diaxial ROESY correlations between H_3_-19 (δ_H_ 0.75), H-2b (δ_H_ 1.48), H-4b (δ_H_ 1.41), and H-11b (δ_H_ 1.42).

A number of overlapping resonances in the ^1^H spectrum made the assignment of relative configuration at other centers difficult. Therefore, comparisons with previously reported plakinamine steroidal alkaloids were conducted. A transfused C/D ring, β-oriented side chain at C-17, and an α-methyl group at C-20 can be observed in all of the plakinamine steroidal alkaloids previously reported. By comparing the ^13^C NMR data for **1** (C-13, δ_C_ 44.7; C-14, δ_C_ 56.3; C-17, δ_C_ 57.2; C-20, δ_C_ 38.2) with the published data for structurally similar plakinamines [5,10,11,13,14], assignment of the relative configuration at these centers was possible as they fall within the ranges typically reported (C-13, δ_C_ 44.0 ± 0.8; C-14, δ_C_ 54.7 ± 1.6; C-17, δ_C_ 57.2 ± 1.6; C-20, δ_C_ 36.5 ± 2.0). The 2D-NOESY and ROESY data are consistent with the assigned structure. Plakinamines L and M are the only other compounds in the class that bear an acyclic side chain [13,14]. Plakinamine P differs due to a change in the position of the double bond from C-23/C-24 in plakinamines L and M to C-24/C-28 in **1**. The *Z* geometry of the double bond was determined based on ROESY correlations observed from H-28 to H-25, H_3_-26, and H_3_-27.

### 2.2. Biological Activity

**Figure 3:**
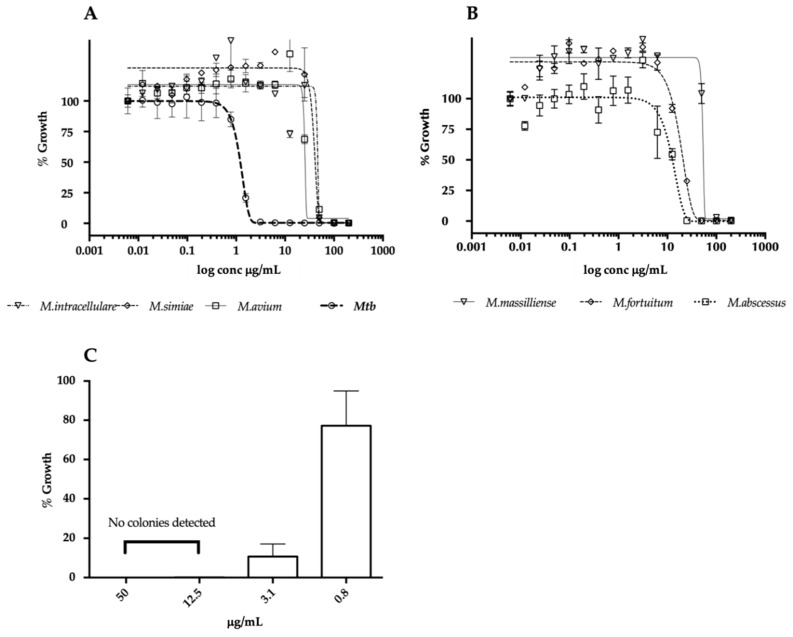
Activity of plakinamine P against multiple mycobacterial pathogens. (**A**,**B**) Cultures were treated with 16-point, 2-fold serial dilutions of plakinamine P for 2 days (fast-growing mycobacteria – panel **B**) and 5 days (slow-growing mycobacteria – panel **A**) after which the luminescence was read. The Gompertz model was used to calculate MIC (99% killing). (**C**) Bactericidal activity of plakinamine P against *Mtb* was evaluated. Cultures treated with 50, 12.5, 3.1 and 0.8 μg/mL plakinamine P were plated on 7H10 OADC and incubated for 3 weeks before colony forming unit (CFU) enumeration. The data are presented as % growth relative to average CFU/mL of the 2% DMSO controls.

In a primary screen, the fraction from the marine organism of the genus *Plakina* sp. (HBOI.010.F07) inhibited actively growing *M. tuberculosis* CDC1551 by 97.4%, and 62% inhibition was detected against nonreplicating dormant bacteria. The data with nonreplicating bacteria were obtained using a multistress dormancy model which includes hypoxia, acidic pH, and starvation as the cues for *Mtb* to stop replicating [3]. Activity against dormant *Mtb* was absent when colony forming units (CFUs) were evaluated [16]. Plakinamine P was identified as the predominant compound in the mixture. Further purification yielded 1 mg of pure compound which did not retain activity against dormant *Mtb*, therefore the dormancy assay was no longer used for this compound. All the microbiological results reported were obtained with auto-luminescent mycobacteria expressing the *luxABCDE* operon as previously described [3]. Dose–response curves were conducted against *Mtb* as well as a panel of opportunistic non-tuberculous mycobacterial (NTM) pathogens, including both rapid-growing NTMs (*M. massilliense*, *M. abscessus*, *M. fortuitum*) and slow-growing NTMs (*M. avium*, *M. intracellulare*, *M. simiae*). All minimum inhibitory concentration (MIC) values are listed in Table 2 and dose–response curves are shown in Figure 3A,B. Plakinamine P was active against all mycobacterial species tested, with the lowest MIC of 1.8 µg/mL observed for *Mtb* and the highest of 57 µg/mL observed for *M. intracellulare*. The IC_50_ against J774 macrophages was 15.2 µg/mL, to produce a selectivity index (SI; IC_50_/MIC) of 8.5 against *Mtb*. The potent bactericidal activity of plakinamine P against *Mtb* was confirmed by plating samples for colony forming units (CFU) enumeration. These results demonstrated 90% bacterial killing by the compound at 3.1 µg/mL against *Mtb*. Furthermore, at the higher concentrations of 12.5 and 50 µg/mL, zero colonies were observed, indicating sterilization of the *Mtb* culture at those concentrations (Figure 3C).

## 3. Discussion

The major global health threat posed by *Mtb* infections is intensified by the difficulty to treat TB. In this study, we have identified a novel scaffold bactericidal against *Mtb* with low toxicity towards mammalian cells. The structural characterization of this compound revealed it to be the novel steroidal alkaloid plakinamine P. Plakinamine P inhibited the growth of *M. tuberculosis* with an MIC of 1.8 µg/ml. It is most closely related to plakinamines L and M, which have been reported to inhibit the growth of *M. tuberculosis* strain H37Ra with MICs of 3.6 and 15.8 µg/mL, respectively [14]. Selectivity for mycobacteria versus mammalian cells and spectrum of activity against mycobacteria has not been reported for the other known plakinamines. In the current study, plakinamine P was most potent against *Mtb*, however, it was also moderately active against important opportunistic mycobacteria including *M. abscessus* and *M. avium* complex organisms. These are highly drug-tolerant pathogens known to cause chronic infections in immunocompromised individuals [18,19]. Nevertheless, the observed selectivity of plakinamine P for *Mtb* in comparison to other mycobacteria suggests it may target pathways uniquely essential for *Mtb* survival.

The delayed clearance of *Mtb* by current front-line TB drugs is often attributed to the distinctive physiological aspects of this intracellular pathogen during infection [20]. In light of this, many TB drug discovery studies are focusing efforts on finding scaffolds capable of inhibiting *Mtb’*s survival pathways during infection [21,22]. Plakinamine P described in this study is structurally similar to cholesterol, with a modified side chain. Previous work has shown the essentiality of cholesterol catabolism for the survival and persistence of *Mtb* during infection [4]. Inhibition of this pathway leads to death of intracellular *Mtb* [23]. Additionally, cholesterol analogs with undegradable side chains are capable of killing *Mtb* in culture [24]. Our data, combined with previously published work, suggest that plakinamine P may be causing mycobacterial death by inhibiting the cholesterol catabolism pathway. Additionally, inhibition may be due to toxic byproducts coming from the breakdown of plakinamine P via the cholesterol degradation pathway.

In conclusion, we have characterized an MNP-derived scaffold with potent antimycobacterial activity. The essentiality of the putative target of plakinamine P for *Mtb* survival and persistence in the host further highlights the potential of this compound in TB drug discovery. Future research will not only focus on confirming the molecular target of plakinamine P, but also demonstrating its activity against *Mtb* under in vivo-like conditions.

## 4. Materials and Methods

### 4.1. Chemical Ananlysis

Optical rotation was measured on a Rudolph Research Analytical AUTOPOL III automatic polarimeter. UV spectra were collected on a NanoDrop Spectrophotometer (Thermo Fisher Scientific, Inc., MA, USA). NMR data were collected on a JEOL ECA-600 spectrometer (JEOL USA, Peabody, MA, USA) operating at 600 MHz for ^1^H, and 150.9 for ^13^C. The edited *g*HSQC spectrum was optimized for 140 Hz and the gHMBC spectrum optimized for 8 Hz. Chemical shifts were referenced to solvent, e.g., CD_3_OD, δ_H_ observed at 3.31 ppm and δ_C_ observed at 49.1 ppm or DMSO- *d*_6_ δ_H_ observed at 2.50 ppm and δ_C_ observed at 39.5 ppm. High resolution mass spectrometry was performed on a JEOL AccuTOF-DART 4G using the DART source for ionization.

#### 4.1.1. Biological Material

The steroidal alkaloid Plakinamine P, **1**, was isolated from a sponge identified as *Plakina* n. sp. (S. 26, a picture of the sponge can be found in Figure S26A) (Phylum Porifera, Class Homoscleromorpha, Order Homosclerophorida, Family Plakinidae). The specimens (HBOI ID; 25-V-93-3-009, HBOI Museum Number 004.0001) were collected at a depth of 93 m using the Johnson Sea-Link I manned submersible in the Bahamas off the NW tip of Crooked Island near Pittstown (latitude 22 49.278’ N, longitude, 74 21.075’W). The external morphology is bulbous to globular (1–2 cm thick, 2–6 cm in diameter), with a single oscula per bulb. Oscules are less than 4 mm wide with a tubular and darkened membrane projection. The surface of the sponge is smooth and the consistency is gelatinous and compressible. The specimen is light brown externally and tan in color internally, both in life and preserved. Spicules are in very low abundance and consist of small ramified calthrops (tetralophose and trilophose), rare smooth non-lophose calthrops, and small diod microrhabs (7–10 µm in length and <1 µm in width). Calthrops are regular in size; less than 20 µm in total size, with rays 8–10 µm × 2–3 µm. Trilophose calthrops usually have deformed rays with variation in the pattern of ramification between rays. The lophose calthrops are typical of the genus *Plakina*. There are five *Plakina* species currently recognized for the Caribbean: *Plakina elisa*, *Plakina nathaliae*, *Plakina tetralopha*, *Plakina jamaicensis*, and *Plakina arletensis* [25]. The present *Plakina* specimen differs from those species in general morphology as it has a much thicker growth, bulbous shape, and unique oscula morphology. The spicule combination, the tendency to have deformed rays and diverse branching patterns within a spicule, and their low density in the body are unique characteristics of this sample that indicate that it may be an undescribed *Plakina* species. Further study of the histology and genetics of this specimen will allow its distinction from the other Caribbean species.

#### 4.1.2. Extraction and Isolation

The sample was frozen immediately after collection and stored at −20 °C until extraction. The frozen sponge (92 g) was soaked overnight in CH_3_OH:H_2_O (1:1 v/v), filtered, and allowed to dry overnight. Extraction of the sponge was accomplished using a Dionex ASE 100^®^ Accelerated Solvent Extractor (Dionex, Sunnyvale, CA, USA) using the following solvents: Extract 1: heptane, Extract 2: ethyl acetate:ethanol (1:1 v/v), Extract 3: ethanol, Extract 4: methanol:water (3:1 v/v) and washed with isopropyl alcohol. The methanol:water (3:1 v/v) extract and the isopropanol extract were combined and separated by medium pressure liquid chromatography using a Teledyne Isco Combiflash^®^ Rf 4x equipped with PeakTrak software (Version 2.1.19, Teledyne Isco, Lincoln, NE, USA) as follows: 365 mg of the combined extract was absorbed onto approximately 2 g of C18 reversed-phase packing, dried, and then placed into a loading column. A Teledyne Isco 15.5 g C18 Redisep Rf Gold column operating at a flow rate of 30 mL/min and monitored at 225 and 280 nm and collected into 13 mm tubes was used for the separation. Solvent A was H_2_O:CH_3_CN (95:5), Solvent B was CH_3_CN, Solvent C was CH_3_OH, and Solvent D was CH_2_Cl_2_. The run lasted 15.9 mins and 37 column volumes. The column was first eluted with a mixture of A:B (94:6 v/v) for 2 column volumes. The column was then eluted over a linear gradient to 100% B over 21 column volumes and then held at 100% B for an additional 3 column volumes. The column was then washed with 100% Solvent C (CH_3_OH), followed by a rapid gradient of CH_2_Cl_2_ in CH_3_OH to wash the column. The anti-mycobacterial activity was observed in Fraction 7 eluting as a broad peak between column volumes 8 to 15 containing > 90% plakinamine P (0.040 g, 0.043% frozen weight). A portion of this material was further fractionated by semi-preparative HPLC at room temperature using a Vydac C18 protein and peptide column (10 × 250 mm, 10 µm particle size) and the following gradient Solvent A: H_2_O:CH_3_CN:TFA (95:5:0.1 v/v/v), and Solvent B: CH_3_CN:TFA (100:0.1 v/v) t = 0 min. A:B 4:1; *t* = 5 min. A:B 1:1, hold for 10 min; *t* = 20 min. 100% B, hold for 8 mins. Plakinamine P elutes at 12.5 mins.

Plakinamine P (1); tan oil; [α]20D = +16.9 (*c* 0.11 in MeOH); UV (MeOH) *λ_max_* (log ε) 195 nm (2.7); ^1^H and ^13^C NMR (Table 1, Figures S15 and S26); DART HRMS: C_33_H_58_N_2_ [*m*/*z* observed 483.4693 [M + H]^+^, calcd. 483.4678, Δ = -1.5 mmu], Figure S25.

### 4.2. Biological Activity

Four slow-growing and 3 fast-growing mycobacterial strains (Table 2) were cultured in Middlebrook 7H9 broth media, supplemented with 0.05% Tween 80, 10% OADC, and 50 µg/mL kanamycin when necessary for plasmid maintenance. The previously described *Mtb*-Lux strain constitutively expressing the *luxCDABEG* operon from the episomal plasmid pMV306hsp+LuxG13, was used [3]. pMV306hsp+LuxG13 was a gift from Brian Robertson & Siouxsie Wiles (Addgene plasmid # 26161; http://n2t.net/addgene:26161; RRID:Addgene_26161). All NTMs were transformed with the same plasmid to produce a stable luminescent signal. Log phase cultures of the mycobacterial strains expressing the *lux* operon were diluted to an OD600 of 0.01 and treated with serial dilutions of plakinamine P. This compound was resuspended in 100% dimethyl-sulfoxide (DMSO) at 10 mg/mL. Sixteen-point 2-fold serial dilutions of plakinamine P, starting at 200 µg/mL, were prepared in a final 2% DMSO concentration, 12 µg/mL rifampicin and 2% DMSO were used as controls. Treatments were performed in 384-well plates for 5 days at 37 °C (5% CO_2_, ambient O_2_, and humidity) for slow-growing mycobacteria and 2 days for fast-growing mycobacteria (Table 1), after which the luminescence was read using Synergy^TM^ H4 plate reader (BioTeK). Samples of *Mtb*-Lux treated with 0.8, 3.1, 12.5 and 50 µg/mL plakinamine P were taken, diluted 10-fold in PBS-Tween80, and plated in 7H10 OADC. Plates were incubated for 3 weeks at 37 °C, after which colonies were enumerated.

J774A.1 (ATCC^®^ TIB67™) macrophages were cultured in Dulbecco’s Modified Eagle Medium (DMEM, GIBCO), supplemented with 10% heat inactivated fetal calf serum (Atlanta Biologicals), 1 mM sodium pyruvate (Mediatech, Inc.), 2mM L-glutamine (Mediatech, Inc.), and 1% PenStrep (100 U/mL Penicillin, 100 µg/mL Streptomycin, GIBCO). Twenty-five thousand cells/well were seeded overnight in black 384-well plates, then treated with serial dilutions of plakinamine P as described above. Tryton X and 2% DMSO were used as controls in these experiments.

Data were normalized to highest and lowest output values in the dose response. MIC (99% killing) and IC_50_ were calculated using a Gompertz model, and a nonlinear regression—normalized response curve fit, respectively in GraphPad Prism 5 [26]. The selectivity index (SI) was calculated as IC_50_/MIC.

## Figures and Tables

**Table 1:** ^1^H and ^13^C NMR data for plakinamine P (**1**) (CD_3_OD, 600 MHz).

Position.	*δ*_C_, Type	*δ*_H_ (*J* in Hz)	COSY	HMBC *^a^*
1a	38.2, CH_2_	2.01 (ddd, 13.8, 4.1, 4.1)	1b, 2ab	3, 5, 10
1b		1.21 (ddd, 13.8, 13.8, 3.4)	1a, 2ab	19
2a	23.7, CH_2_	1.95 (m)	1ab, 2b, 3	1, 3, 4, 10
2b		1.62 (m)	1ab, 2a, 3	1, 3, 10
3	66.8, CH	3.21 (m)	2ab, 4ab	
4a	29.7, CH_2_	1.84 (m)	3, 4b, 5	2, 3, 10
4b		1.51 (m)	3, 4a, 5	3, 5
5	41.9, CH	1.51 (m)	4ab, 6ab	3
6a	30.6, CH_2_	1.86 (m)	5, 7	7, 8
6b		1.30 (m)	5, 7	4, 5
7	118.4, CH	5.20 (br d, 2.8)	6ab	5, 6, 9, 14
8	140.7, C			
9	50.5, CH	1.73 (m)	11ab	8, 11
10	35.5, C			
11a	22.7, CH_2_	1.63 (m)	9, 11b, 12ab	8, 9
11b		1.52 (m)	9, 11a, 12ab	8, 9, 10, 13
12a	40.9, CH_2_	2.09 (m)	11ab, 12b	9, 11, 13, 14
12b		1.28 (m)	11ab, 12a	
13	44.7, C			
14	56.3, CH	1.87 (m)	15ab	7, 8, 13
15a	24.2, CH_2_	1.57 (m)	14, 15b, 16ab	16
15b		1.46 (m)	14, 15a, 16ab	
16a	29.2, CH_2_	1.93 (m)	15ab, 16b, 17	13, 15
16b		1.33 (m)	15ab, 16a, 17	15, 17, 20
17	57.2, CH	1.29 (m)	16ab, 20	12, 13, 16, 18, 20, 21
18	12.4, CH_3_	0.59 (s)		12, 13, 14, 17
19	13.4, CH_3_	0.86 (s)		1, 5, 9, 10
20	38.2, CH	1.45 (m)	17, 21, 22ab	22, 23
21	19.4, CH_3_	1.05 (d, 6.9)	20	17, 20
22a	36.8, CH_2_	1.46 (m)	20, 22b, 23ab	
22b		1.17 (m)	20, 22a, 23ab	20, 21
23a	28.1, CH_2_	2.24 (ddd, 12.7, 12.7, 4.8)	22ab, 23b	22, 24, 25, 28
23b		2.07 (m)	22ab, 23a	22, 24, 25, 28
24	159.9, C			
25	36.1, CH	2.38 (sep, 6.9)	26, 27	23, 24, 26/27, 28
26	22.6, CH_3_	1.09 (d, 6.9)	25	24, 25, 27
27	22.4, CH_3_	1.10 (d, 6.9)	25	24, 25, 26
28	111.7, CH	5.31 (t, 6.9)	29ab	23, 24, 25, 29
29	56.4, CH_2_	3.76 (dd, 7.6, 2.1)	28, 29b	24, 28, 30/31
30	42.7, CH_3_	2.84 (s)		29, 30/31
31	42.7, CH_3_	2.84 (s)		29, 30/31
32	40.5, CH_3_	2.85 (s)		3, 32/33
33	40.5, CH_3_	2.85 (s)		3, 32/33

*^a^g*HMBC correlations, optimized for 8 Hz, are from proton(s) stated to the carbons listed.

**Table 2:** Species used in this study and corresponding plakinamine P activity.

Plasmid	Source	ID	MIC (µg/mL)
*pMV306hsp+LuxG13*	Addgene plasmid #26161 [17]	N/A	
Fast-growing mycobacteria			
*M. massilliense*		*Mma*	57.6
*M. fortuitum*		*Mfo*	32.45
*M. abscessus*		*Mab*	22.16
Slow-growing mycobacteria			
*M. tuberculosis*	[3]	*Mtb*	1.84
*M. avium*		*Mav*	27.28
*M. intracellulare*		*Min*	49.53
*M. simiae*		*Msi*	48.25

N/A, non applicable. *Rifampicin MIC 0.01 µg/mL/Isoniazid MIC 0.04 µg/mL.

## Supplementary Materials

The following are available online at https://www.mdpi.com/1660-3397/17/12/707/s1, Figure S1: ^1^H NMR (600 MHz, CD_3_OD) of Plakinamine P (**1**), Figure S2: Expansion of ^1^H NMR (600 MHz, CD_3_OD) of Plakinamine P (**1**), Figure S3: ^13^C NMR (150 MHz, CD_3_OD) of Plakinamine P (**1**), Figure S4: gDQF-COSY spectrum (600 MHz, CD_3_OD) of Plakinamine P (**1**), Figure S5: gCOSY spectrum expansion with key side chain correlations (600 MHz, CD_3_OD) of Plakinamine P (**1**), Figure S6: edited gHSQC spectrum (600 MHz, CD_3_OD) of Plakinamine P (**1**), Figure S7: Expansion 1 of edited gHSQC spectrum (600 MHz, CD_3_OD) Plakinamine P (**1**), Figure S8: Expansion 2 of edited gHSQC spectrum (600 MHz, CD_3_OD) of Plakinamine P (**1**), Figure S9: gHMBC spectrum (600 MHz, CD_3_OD) of Plakinamine P (**1**), Figure S10: gHMBC spectrum expansion of N,N dimethyl amino proton correlations (600 MHz, CD_3_OD) of Plakinamine P (**1**), Figure S11: gHMBC spectrum expansion with key side chain correlations (600 MHz, CD_3_OD) of Plakinamine P (**1**), Figure S12: Structure of plakinamine P (**1**) with key gHMBC correlations observed in CD_3_OD (600 MHz), Figure S13: gROESY spectrum (600 MHz, CD_3_OD) of Plakinamine P (**1**), Figure S14: Structure of plakinamine P (**1**) with chemical shifts in CD_3_OD (600 MHz), Figure S15: ^1^H NMR (600 MHz, DMSO-*d*_6_) of Plakinamine P (**1**), Figure S16: Expansion of ^1^H NMR (600 MHz, DMSO-*d*_6_) of Plakinamine P (**1**), Figure S17: ^13^C NMR (150 MHz, DMSO-*d*_6_) of Plakinamine P (**1**), Figure S18: gDQF-COSY spectrum (600 MHz, DMSO-*d*_6_) of Plakinamine P (**1**), Figure S19: edited gHSQC spectrum (600 MHz, DMSO-*d*_6_) of Plakinamine P (**1**), Figure S20: gHMBC spectrum (600 MHz, DMSO-*d*_6_) of Plakinamine P (**1**), Figure S21: NOESY spectrum (600 MHz, DMSO-*d*_6_) of Plakinamine P (**1**), Figure S22: Expansion of Plakinamine P (**1**) 2D-NOESY spectrum (600 MHz, DMSO-*d*_6_) showing key correlations for H-3. Figure S23: ROESY spectrum (600 MHz, DMSO-*d*_6_) of Plakinamine P (**1**), Figure S24: Structure of plakinamine P (**1**) with chemical shifts observed in DMSO-*d*_6_, Figure S25: High resolution DART mass spectrometry data of plakinamine P (**1**), Figure S26: Picture and taxonomic description of the *Plakina* sp. used in the study; Table S1: Table of ^1^H and ^13^C NMR Data for plakinamine P (**1**) (600 MHz, CD_3_OD), Table S2: Table of ^1^H and ^13^C NMR Data for plakinamine P (**1**) (600 MHz, DMSO-*d*_6_).
