# Supplementary material for: Plakinamine P, A Steroidal Alkaloid with Bactericidal Activity against Mycobacterium tuberculosis
Source: Mar Drugs. 2019 Dec 16;17(12):707. doi: 10.3390/md17120707 (PMC6950683; doi:10.3390/md17120707)
Supplement: Supplementary file 1 [file marinedrugs-17-00707-s001.pdf]

Plakinamine P, A Steroidal Alkaloid with Bactericidal Activity against *Mycobacterium tuberculosis*

Carolina Rodrigues Felix [1], Jill C. Roberts [2], Priscilla L. Winder [2], Rashmi Gupta [3], M. Cristina Diaz [2], Shirley A. Pomponi [2], Amy E. Wright [2,*] and Kyle H. Rohde [3,*]

[1] Biotechnology Department, Universidade Federal de Pelotas, Pelotas, RS 96010-610, Brazil.; carolinarodriguesfelix@gmail.com

[2] Harbor Branch Oceanographic Institute, Florida Atlantic University, Fort Pierce, FL 34946, USA; jrober90@fau.edu (J.C.R.); PWINDER@fau.edu (P.L.W.); taxochica@gmail.com (M.C.D.); spomponi@fau.edu

[3] Division of Immunity and Pathogenesis, Burnett School of Biomedical Sciences, College of Medicine, University of Central Florida, Orlando, FL 32827, USA.; rashmi.gupta@ucf.edu

* Correspondence: awrigh33@fau.edu (A.E.W.); kyle.rohde@ucf.edu (K.H.R.); Tel.: +1 772-242-2459 (A.E.W.); +1 407-266-7124 (K.H.R.)

Received: 20 November 2019; Accepted: 10 December 2019; Published: Date

Supporting Information

Figure S1: ^{1}H NMR (600 MHz, CD_3OD) of Plakinamine P (**1**)

Figure S2: Expansion of ^{1}H NMR (600 MHz, CD_3OD) of Plakinamine P (**1**)

Figure S3: ^{13}C NMR (150 MHz, CD_3OD) of Plakinamine P (**1**)

Figure S4: gCOSY spectrum (600 MHz, CD_3OD) of Plakinamine P (**1**)

Figure S5: gCOSY spectrum expansion key side chain correlations (600 MHz, CD_3OD) of Plakinamine P (**1**)

Figure S6: edited gHSQC spectrum (600 MHz, CD_3OD) of Plakinamine P (**1**)

Figure S7: Expansion 1 of edited gHSQC spectrum (600 MHz, CD_3OD) Plakinamine P (**1**)

Figure S8: Expansion 2 of edited gHSQC spectrum (600 MHz, CD_3OD) of Plakinamine P (**1**)

Figure S9: gHMBC spectrum (600 MHz, CD_3OD) of Plakinamine P (**1**)

Figure S10: gHMBC spectrum expansion of N,N dimethyl amino proton correlations (600 MHz, CD_3OD) of Plakinamine P (**1**)

Figure S11: gHMBC spectrum expansion with key side chain correlations (600 MHz, CD_3OD) of Plakinamine P (**1**)

Figure S12: Structure of plakinamine P (**1**) with key gHMBC correlations observed in CD_3OD (600 MHz)

Figure S13: gROESY spectrum (600 MHz, CD_3OD) of Plakinamine P (**1**)

Figure S14: Structure of plakinamine P (**1**) with chemical shifts in CD_3OD (600 MHz)

Table S1: Table of ^{1}H and ^{13}C NMR Data for plakinamine P (**1**) (600 MHz, CD_3OD)

Figure S15: ^{1}H NMR (600 MHz, DMSO-d_6) of Plakinamine P (**1**)

Figure S16: Expansion of ^{1}H NMR (600 MHz, DMSO-d_6) of Plakinamine P (**1**)

Figure S17: ^{13}C NMR (150 MHz, DMSO-d_6) of Plakinamine P (**1**)

Figure S18: gDQF-COSY spectrum (600 MHz, DMSO-d_6) of Plakinamine P (**1**)

Figure S19: edited gHSQC spectrum (600 MHz, DMSO-d_6) of Plakinamine P (**1**)

Figure S20: gHMBC spectrum (600 MHz, DMSO-d_6) of Plakinamine P (**1**)

Figure S21: NOESY spectrum (600 MHz, DMSO-d_6) of Plakinamine P (**1)**

Figure S22: Expansion of Plakinamine P (**1**) 2D-NOESY spectrum (600 MHz, DMSO-d_6) showing key correlations for H-3

Figure S23: ROESY spectrum (600 MHz, DMSO-d_6) of Plakinamine P (**1**)

Figure S24: Structure of plakinamine P (**1**) with chemical shifts observed in DMSO-d_6

Table S2: Table of ^{1}H and ^{13}C NMR Data for plakinamine P (**1) (**600 MHz, DMSO-d_6)

Figure S25: High resolution DART mass spectrometry data of plakinamine P (**1**)

Figure S26: Picture and taxonomic description of the *Plakina* sp. used in the study

S1 ^{1}H NMR (600 MHz, CD_3OD) of Plakinamine P (**1**)

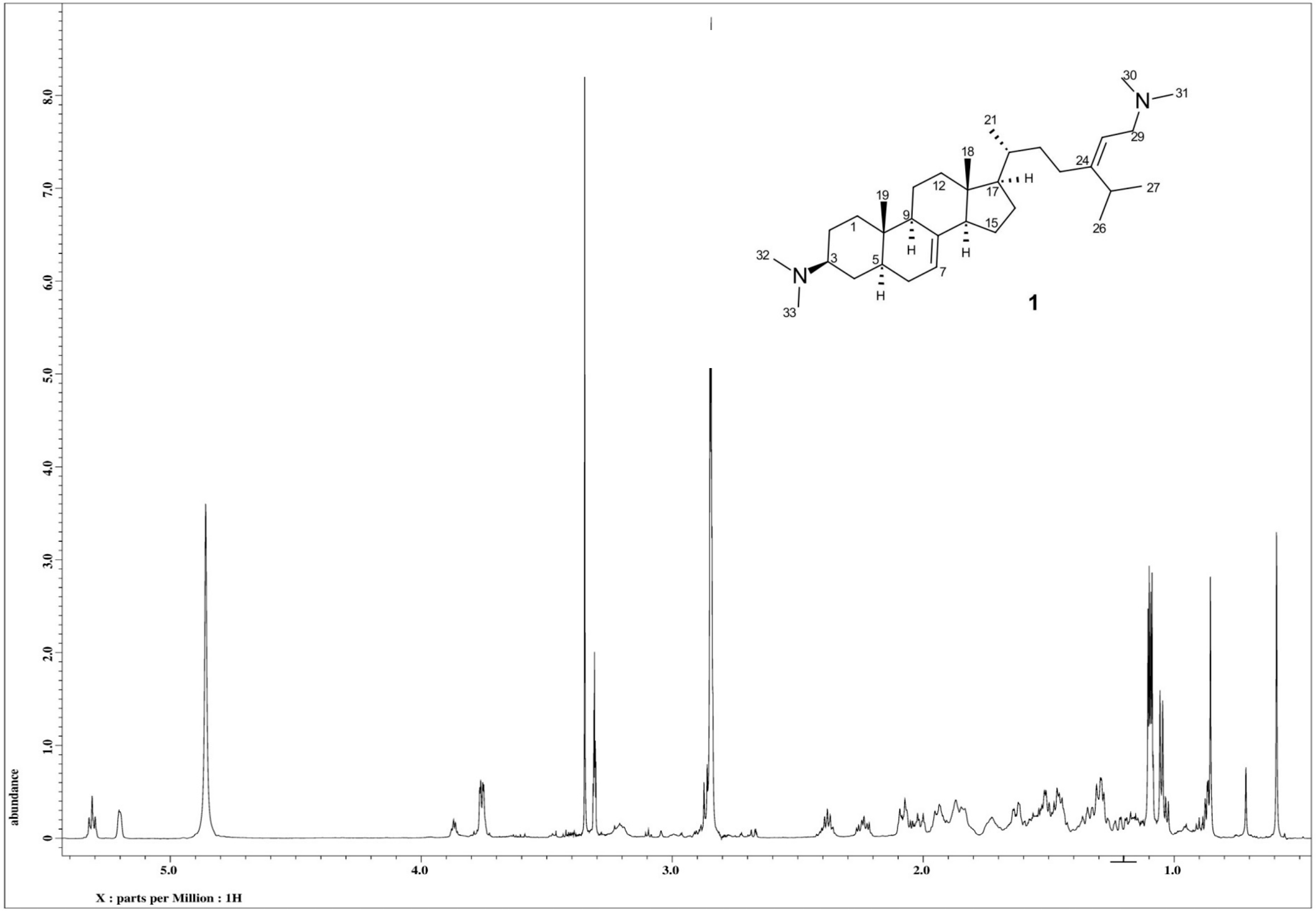

S2 Expansion of ^{1}H NMR (600 MHz, CD_3OD) of Plakinamine P (**1**)

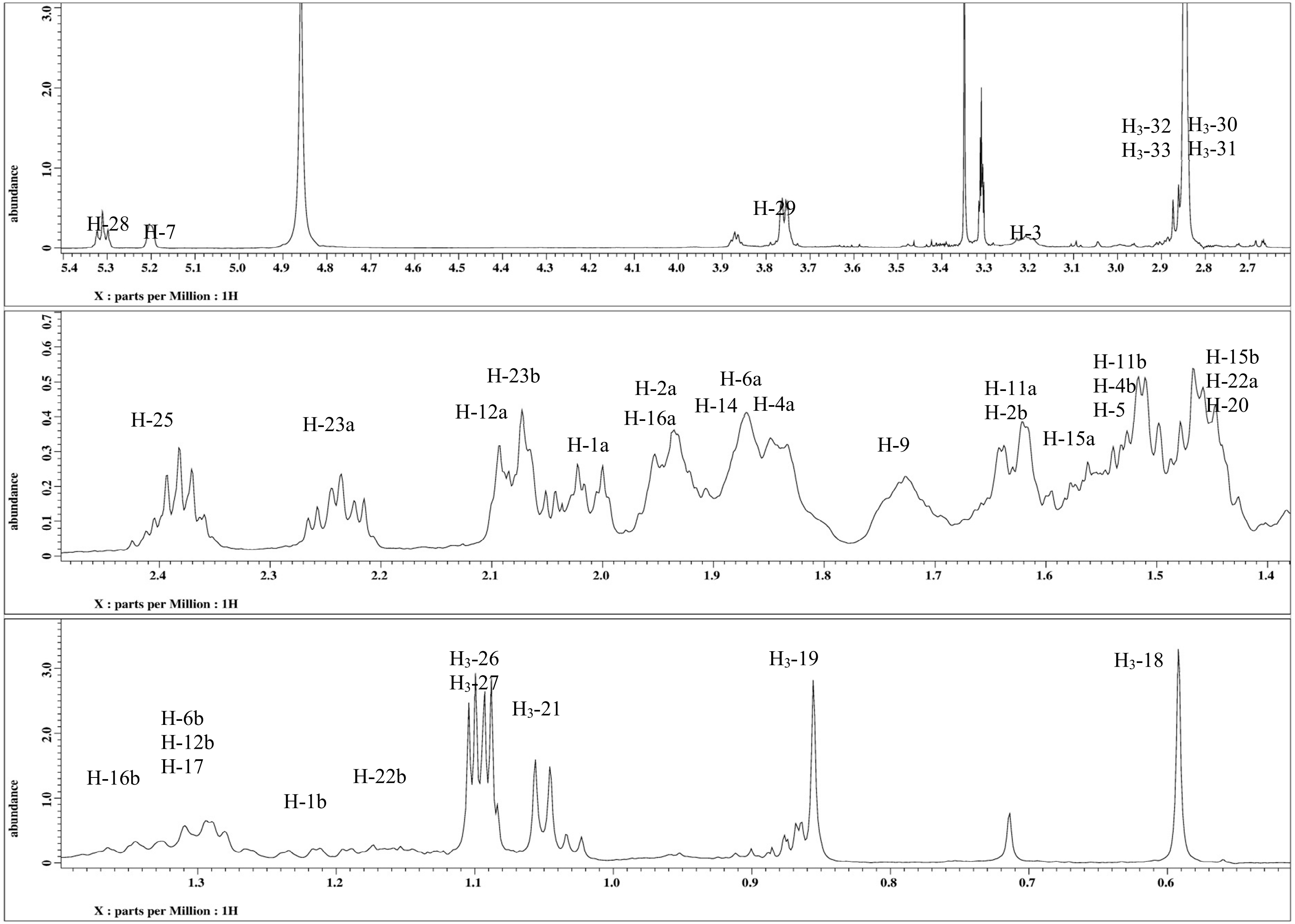

S3 ^{13}C NMR (150 MHz, CD_3OD) of Plakinamine P (**1**)

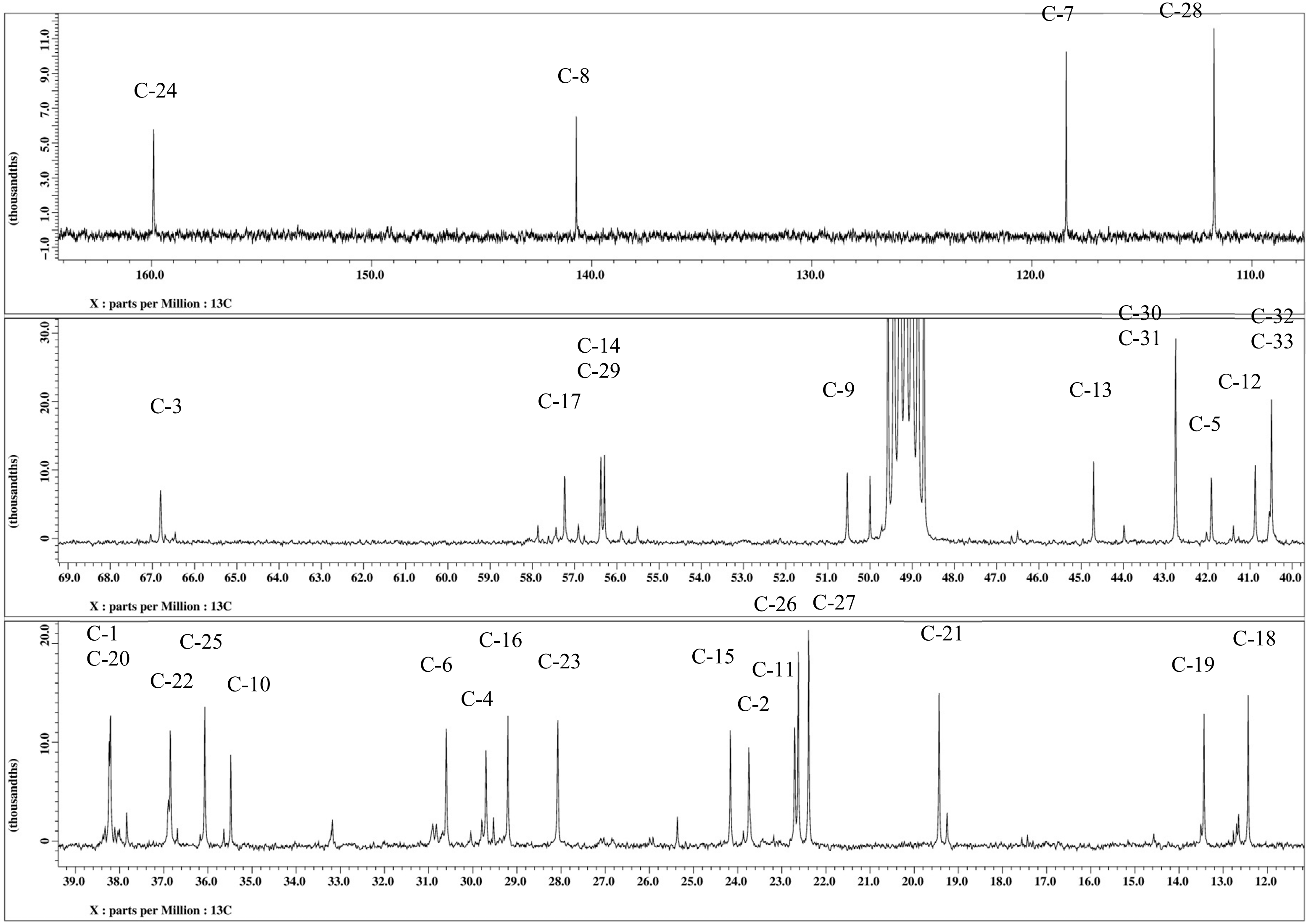

S4 g-COSY spectrum (600 MHz, CD_3OD) of Plakinamine P (**1**)

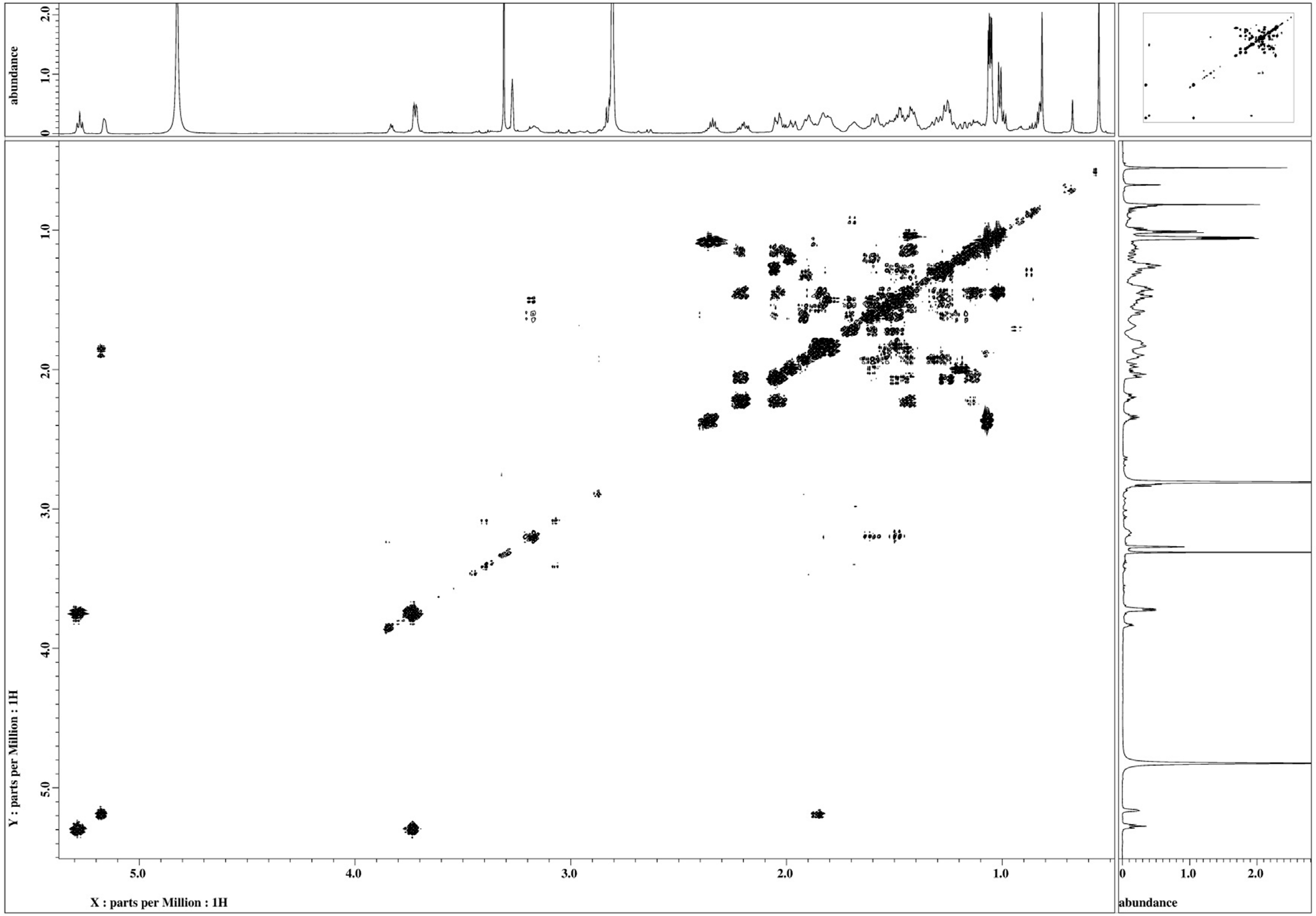

S5 g-COSY spectrum expansion key side chain correlations (600 MHz, CD_3OD) of Plakinamine P (**1**)

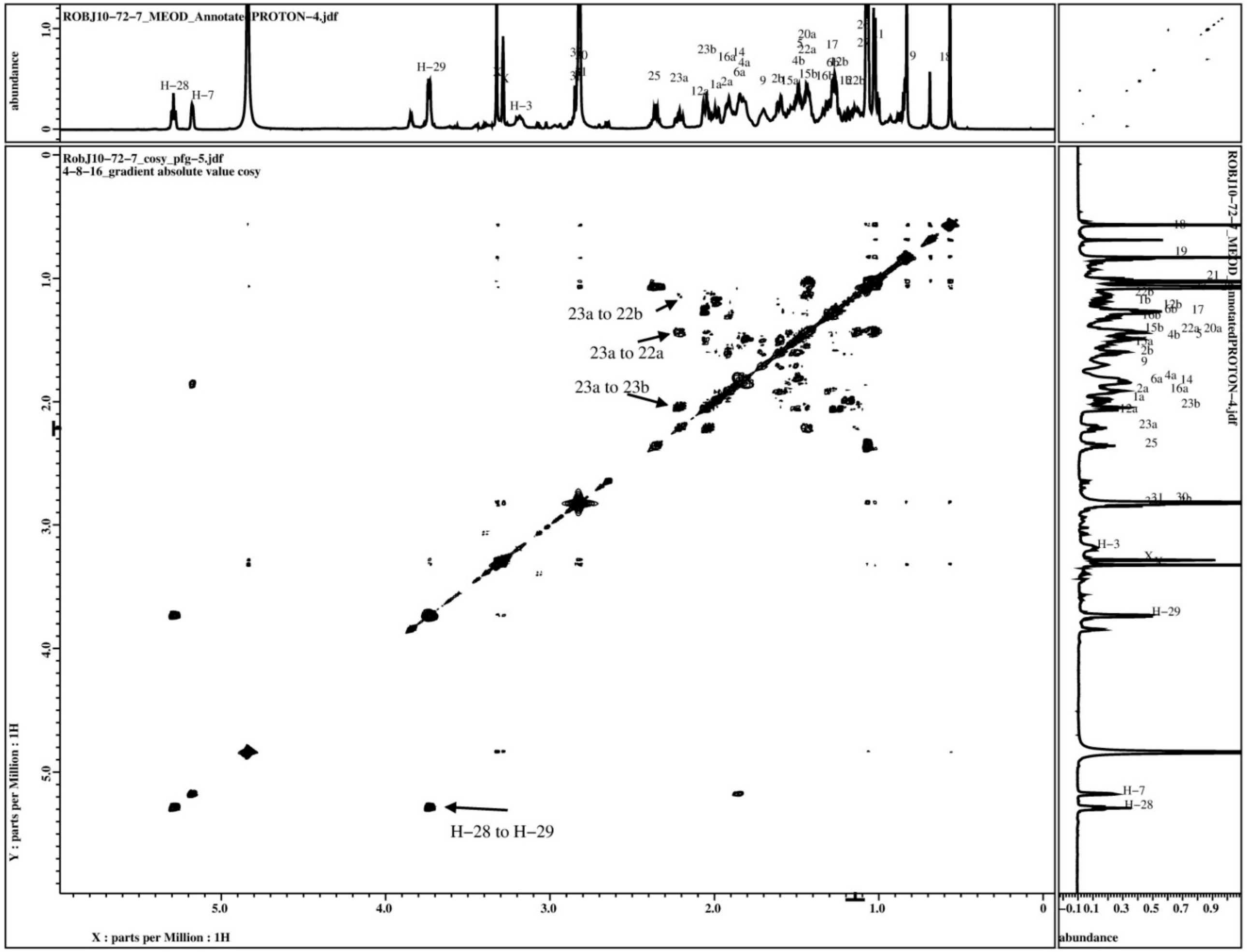

S6 edited gHSQC spectrum (600 MHz, CD_3OD) of Plakinamine P (**1**)

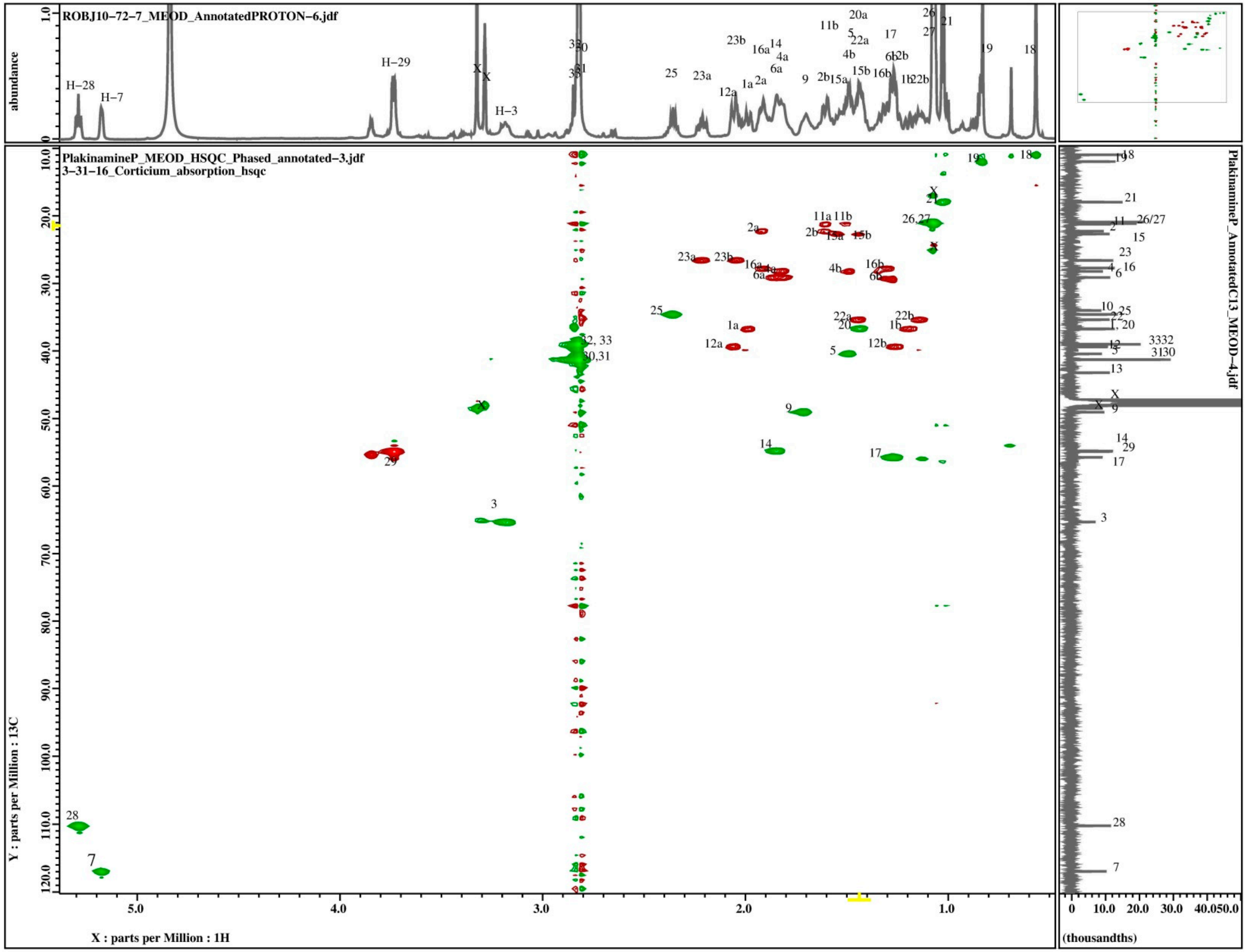

S7 Expansion 1 of edited gHSQC spectrum (600 MHz, CD_3OD) Plakinamine P (**1**)

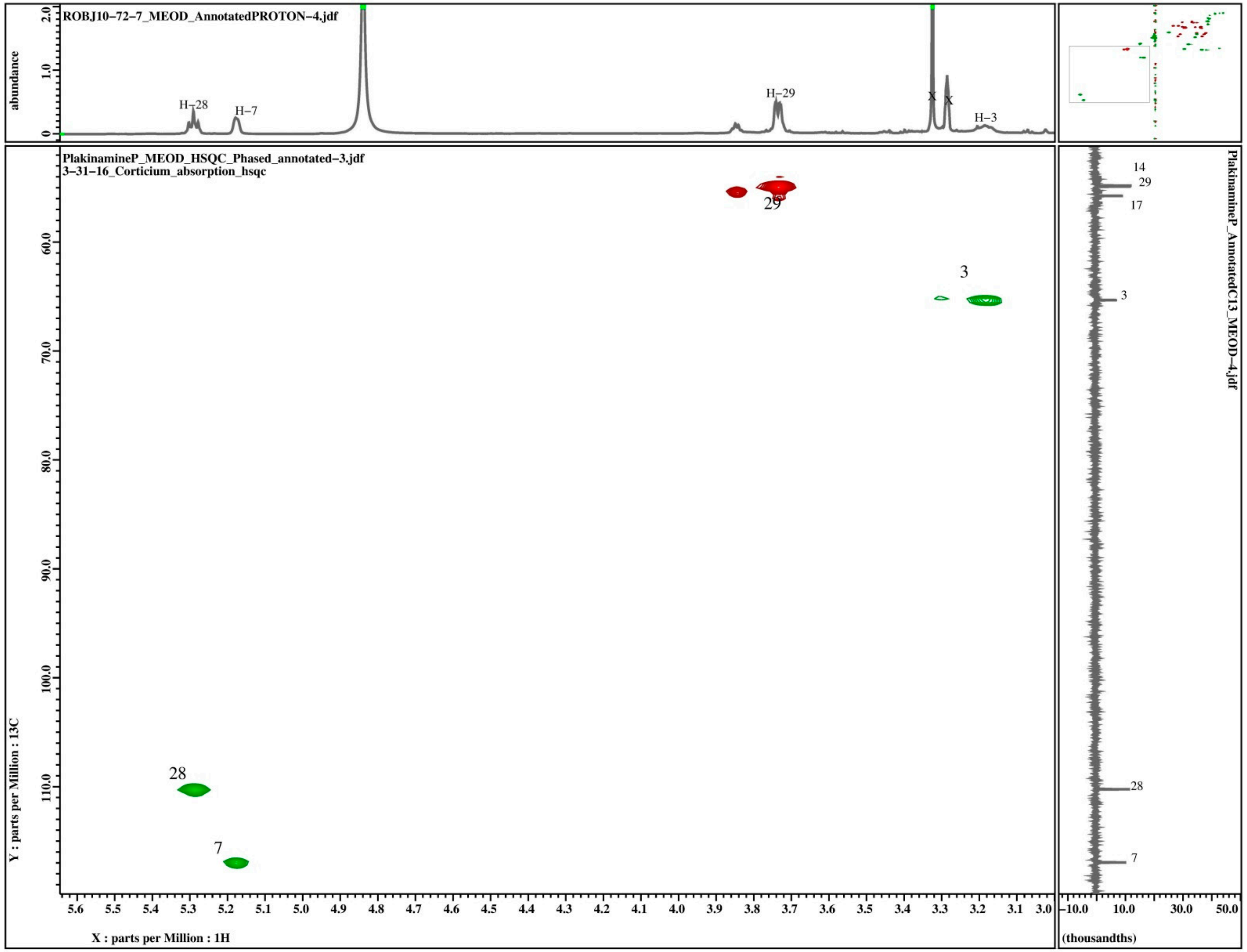

S8 Expansion 2 of edited gHSQC spectrum (600 MHz, CD_3OD) of Plakinamine P (**1**)

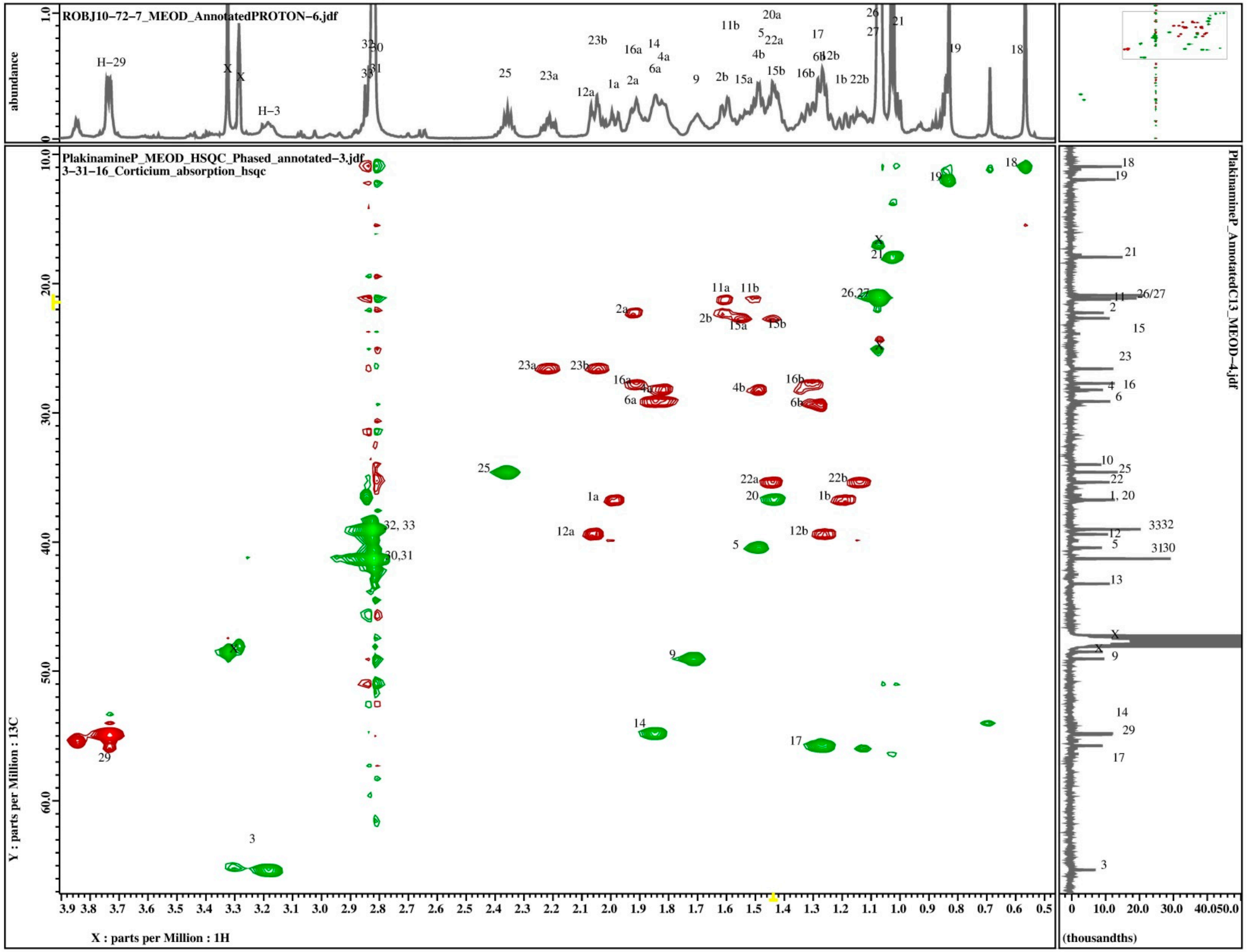

S9 gHMBC spectrum (600 MHz, CD_3OD) of Plakinamine P (**1**)

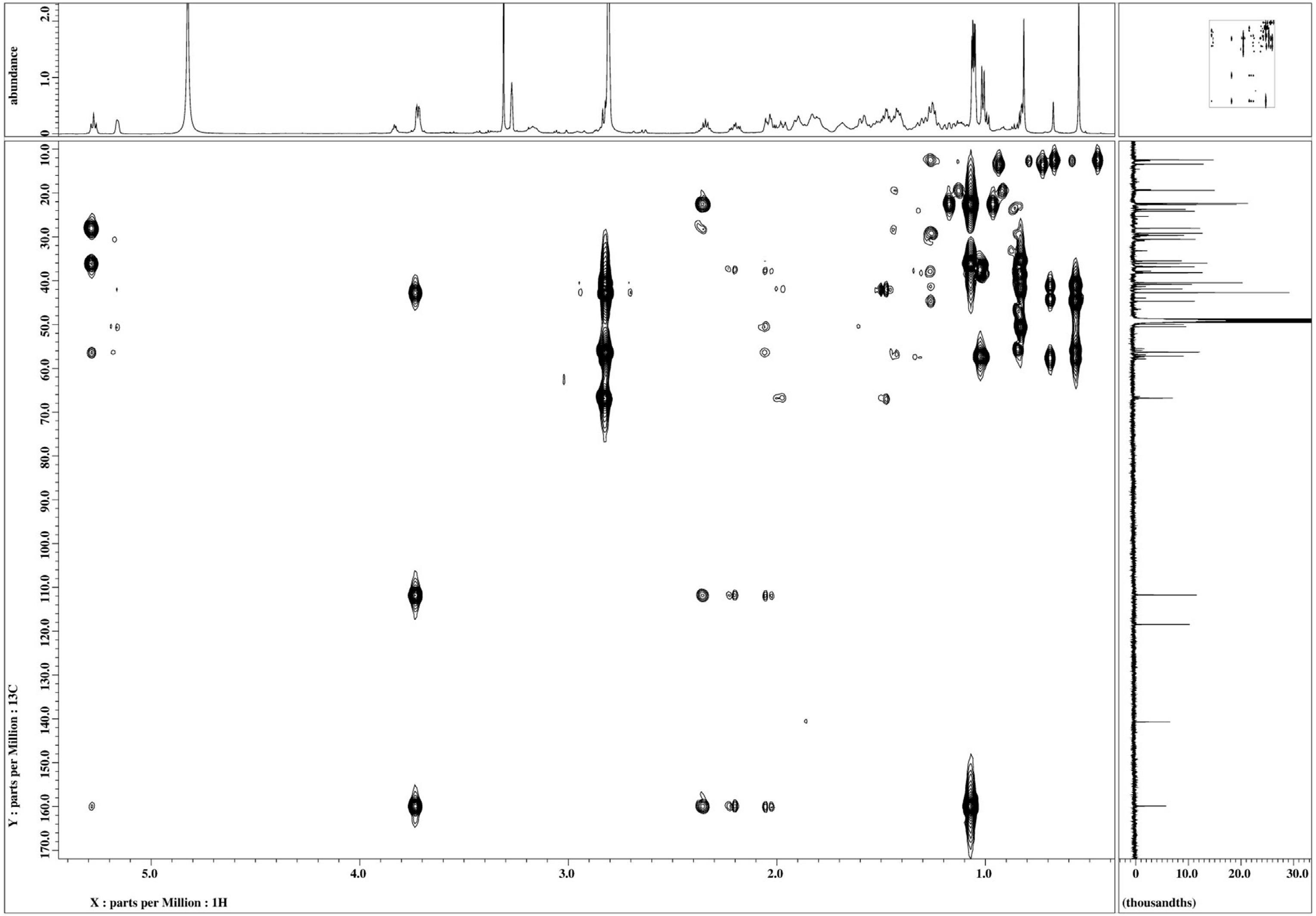

S10 gHMBC spectrum expansion of N,N dimethyl amino proton correlations (600 MHz, CD_3OD) of Plakinamine P (**1**)

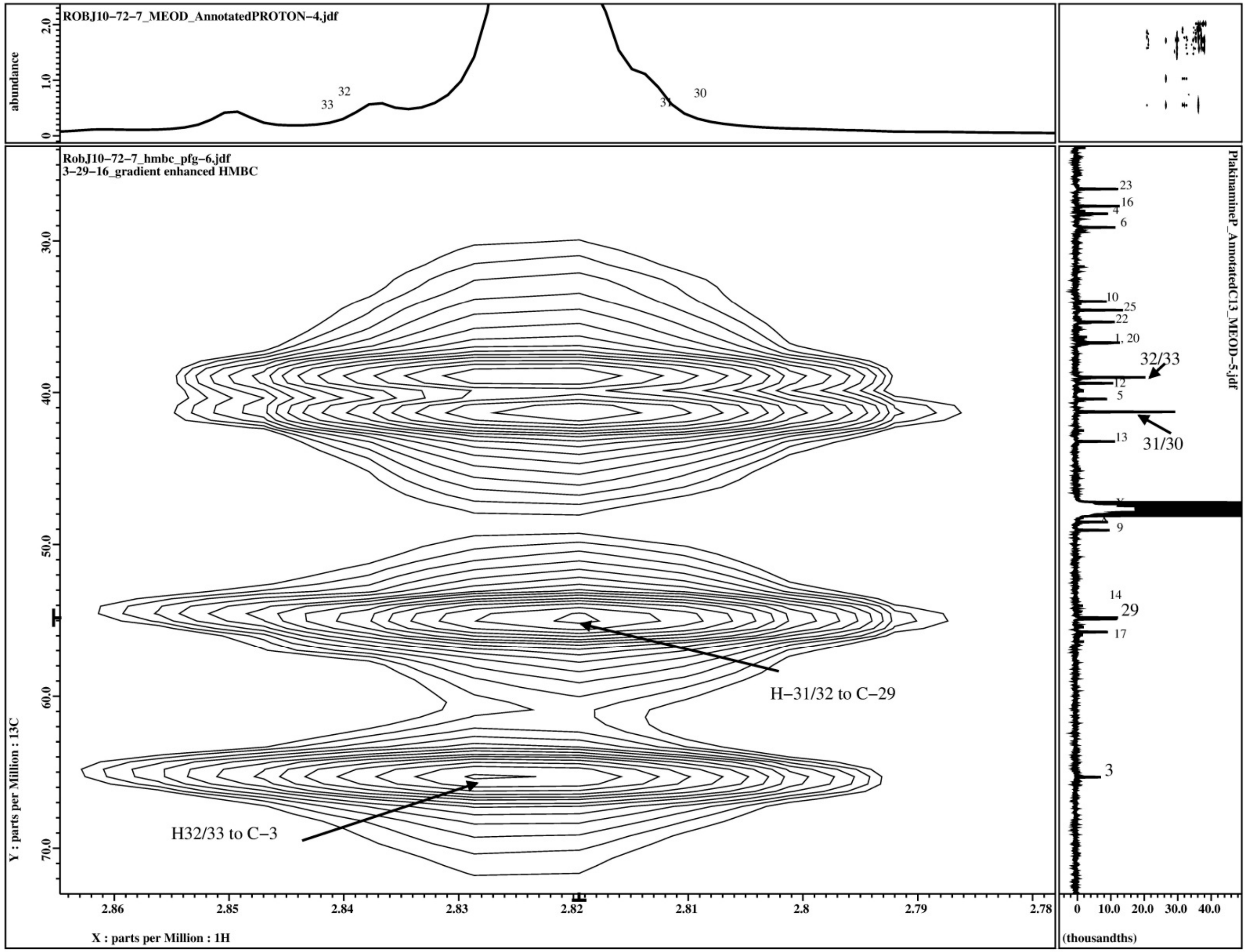

S11 gHMBC spectrum expansion with key side chain correlations (600 MHz, CD_3OD) of Plakinamine P (**1**)

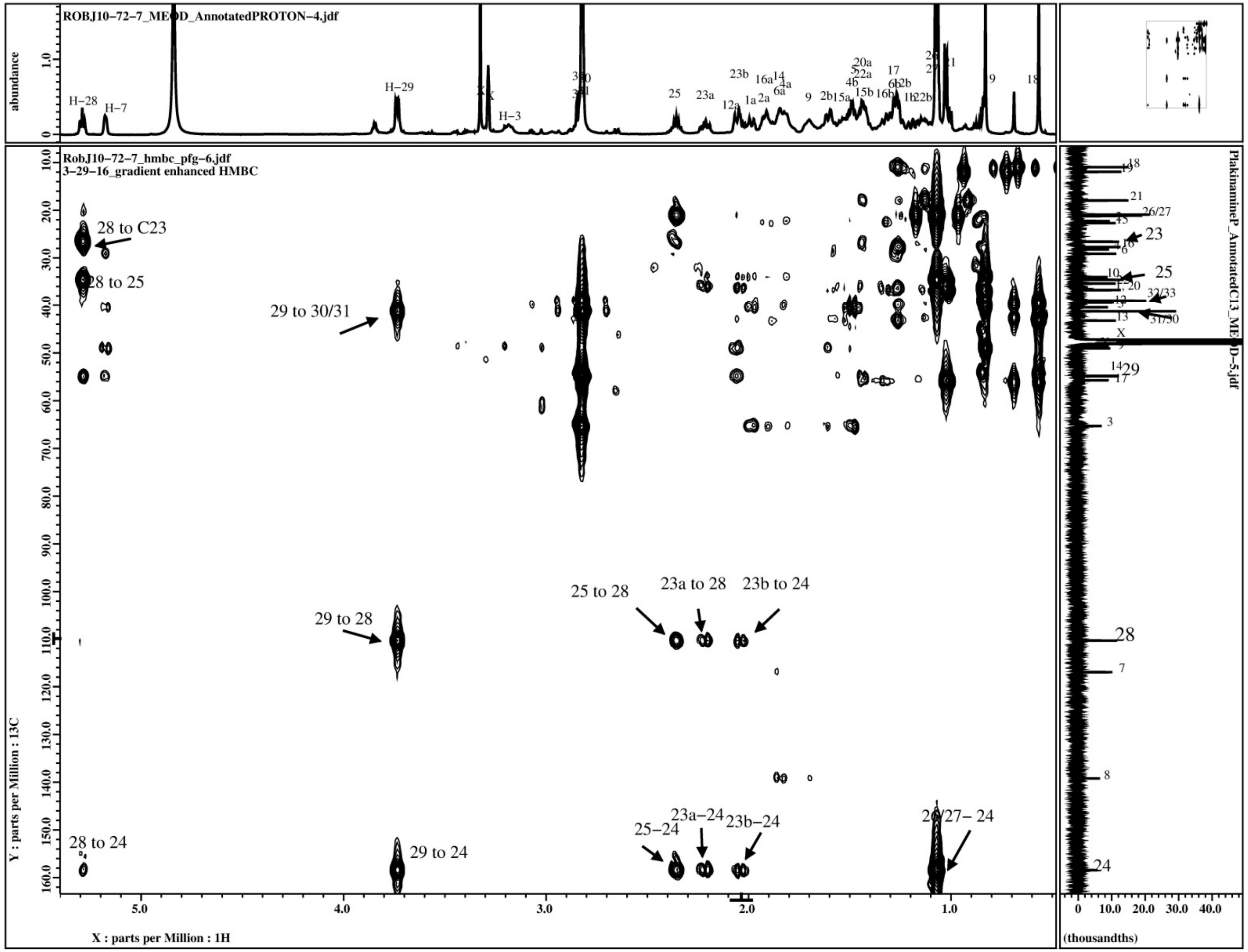

S12 Structure of plakinamine P (**1**) with key gHMBC correlations observed in CD_3OD (600 MHz)

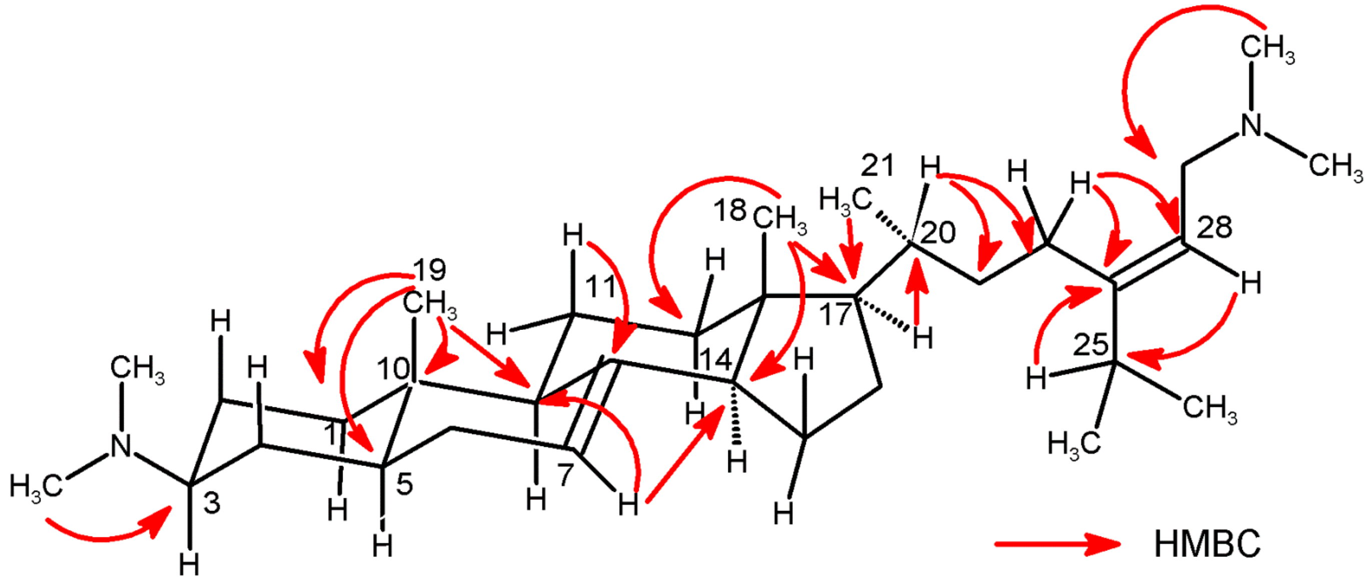

S13 gROESY spectrum (600 MHz, CD_3OD) of Plakinamine P (**1**)

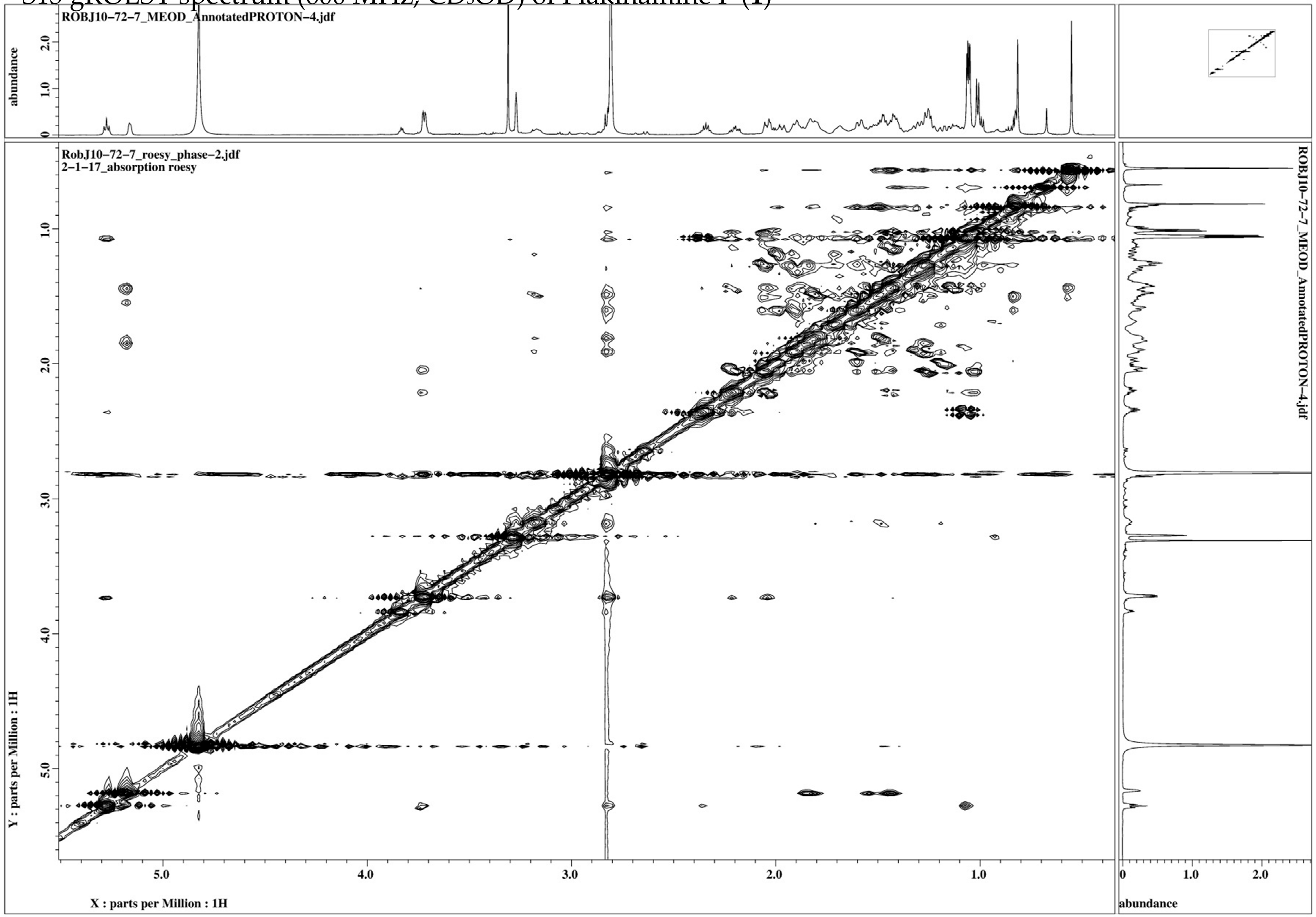

S14 Structure of plakinamine P (**1**) with chemical shifts in CD_3OD (600 MHz)

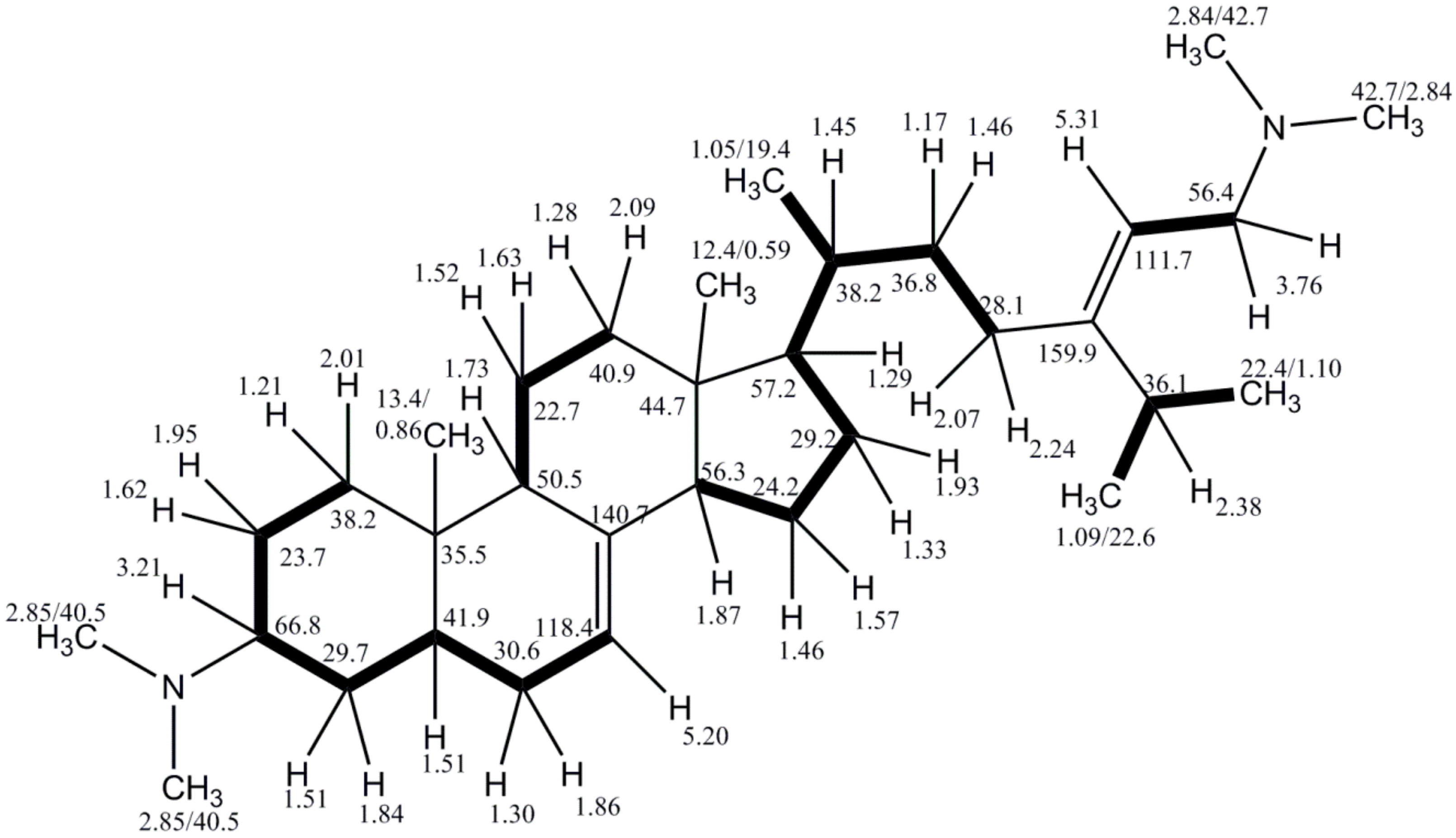

Table S1: Table of ^{1}H and ^{13}C NMR Data for plakinamine P (**1**) (600 MHz, CD_3OD)

position	δ_C, type	δ_H (*J* in Hz)	COSY	HMBC[a]	ROESY
1a	38.2, CH_2	2.01 (ddd, 13.8, 4.1, 4.1)	1b, 2ab	3, 5, 10	1b, 2b
1b		1.21 (ddd, 13.8, 13.8, 3.4)	1a, 2ab	19	1a, 2a, 3, 9
2a	23.7, CH_2	1.95 (m)	1ab, 2b, 3	1, 3, 4, 10	2b, 32/33
2b		1.62 (m)	1ab, 2a, 3	1, 3, 10	1a, 2a, 32/33
3	66.8, CH	3.21 (m)	2ab, 4ab		1b, 32/33
4a	29.7, CH_2	1.84 (m)	3, 4b, 5	2, 3, 10	32/33
4b		1.51 (m)	3, 4a, 5	3, 5	
5	41.9, CH	1.51 (m)	4ab, 6ab	3	
6a	30.6, CH_2	1.86 (m)	5, 7	7, 8	7
6b		1.30 (m)	5, 7	4, 5	
7	118.4, CH	5.20 (br d, 2.8)	6ab	5, 6, 9, 14	14
8	140.7, C				
9	50.5, CH	1.73 (m)	11ab	8, 11	1b, 11a
10	35.5, C				
11a	22.7, CH_2	1.63 (m)	9, 11b, 12ab	8, 9	9, 12a
11b		1.52 (m)	9, 11a, 12ab	8, 9, 10, 13	11a, 12a, 19
12a	40.9, CH_2	2.09 (m)	11ab, 12b	9, 11, 13, 14	11ab, 12b, 18, 21
12b		1.28 (m)	11ab, 12a		11ab, 12a
13	44.7, C				
14	56.3, CH	1.87 (m)	15ab	7, 8, 13	7, 12b
15a	24.2, CH_2	1.57 (m)	14, 15b, 16ab	16	7, 16a, 17
15b		1.46 (m)	14, 15a, 16ab		
16a	29.2, CH_2	1.93 (m)	15ab, 16b, 17	13, 15	15a, 17
16b		1.33 (m)	15ab, 16a, 17	15, 17, 20	16a
17	57.2, CH	1.29 (m)	16ab, 20	12, 13, 16, 18, 20, 21	12a, 14, 16a
18	12.4, CH_3	0.59 (s)		12, 13, 14, 17	12a
19	13.4, CH_3	0.86 (s)		1, 5, 9, 10	11ab
20	38.2, CH	1.45 (m)	17, 21, 22ab		
21	19.4, CH_3	1.05 (d, 6.9)	20	17, 20	12a, 23ab
22a	36.8, CH_2	1.46 (m)	20, 22b, 23ab		
22b		1.17 (m)	20, 22a, 23ab	20, 21	
23a	28.1, CH_2	2.24 (ddd, 12.7, 12.7, 4.8)	22ab, 23b	22, 24, 25, 28	21, 22b, 23b, 29
23b		2.07 (m)	22ab, 23a	22, 24, 25, 28	17, 20, 21, 23a, 29ab
24	159.9, C				
25	36.1, CH	2.38 (sep, 6.9)	26, 27	23, 24, 26/27, 28	26/27, 28
26	22.6, CH_3	1.09 (d, 6.9)	25	24, 25, 27	25, 28
27	22.4, CH_3	1.10 (d, 6.9)	25	24, 25, 26	25, 28
28	111.7, CH	5.31 (t, 6.9)	29ab	23, 24, 25, 29	25, 26/27, 29, 30/31
29	56.4, CH_2	3.76 (dd, 7.6, 2.1)	28, 29b	24, 28, 30/31	23b, 26/27, 28, 30/31
30	42.7, CH_3	2.84 (s)		29, 30/31	28, 29
31	42.7, CH_3	2.84 (s)		29, 30/31	28, 29
32	40.5, CH_3	2.85 (s)		3, 32/33	2b, 3
33	40.5, CH_3	2.85 (s)		3, 32/33	2b, 3

[a]*g*HMBC correlations, optimized for 8 Hz, are from proton(s) stated to the carbons listed

S15 ^{1}H NMR (600 MHz, DMSO-*d*$_6$) of Plakinamine P (**1**)

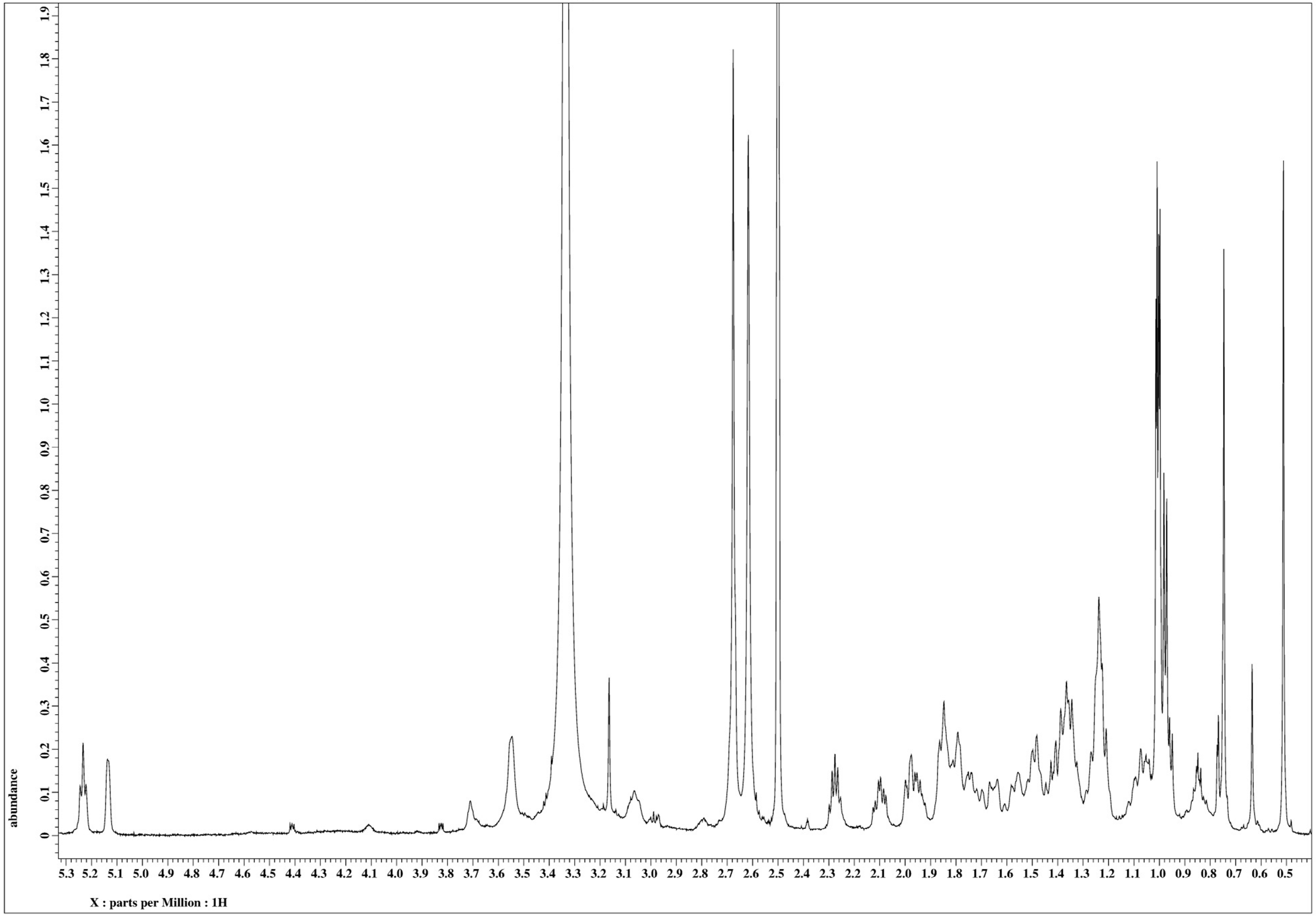

S16 Expansion of ^{1}H NMR (600 MHz, DMSO-d_6) of Plakinamine P (**1**)

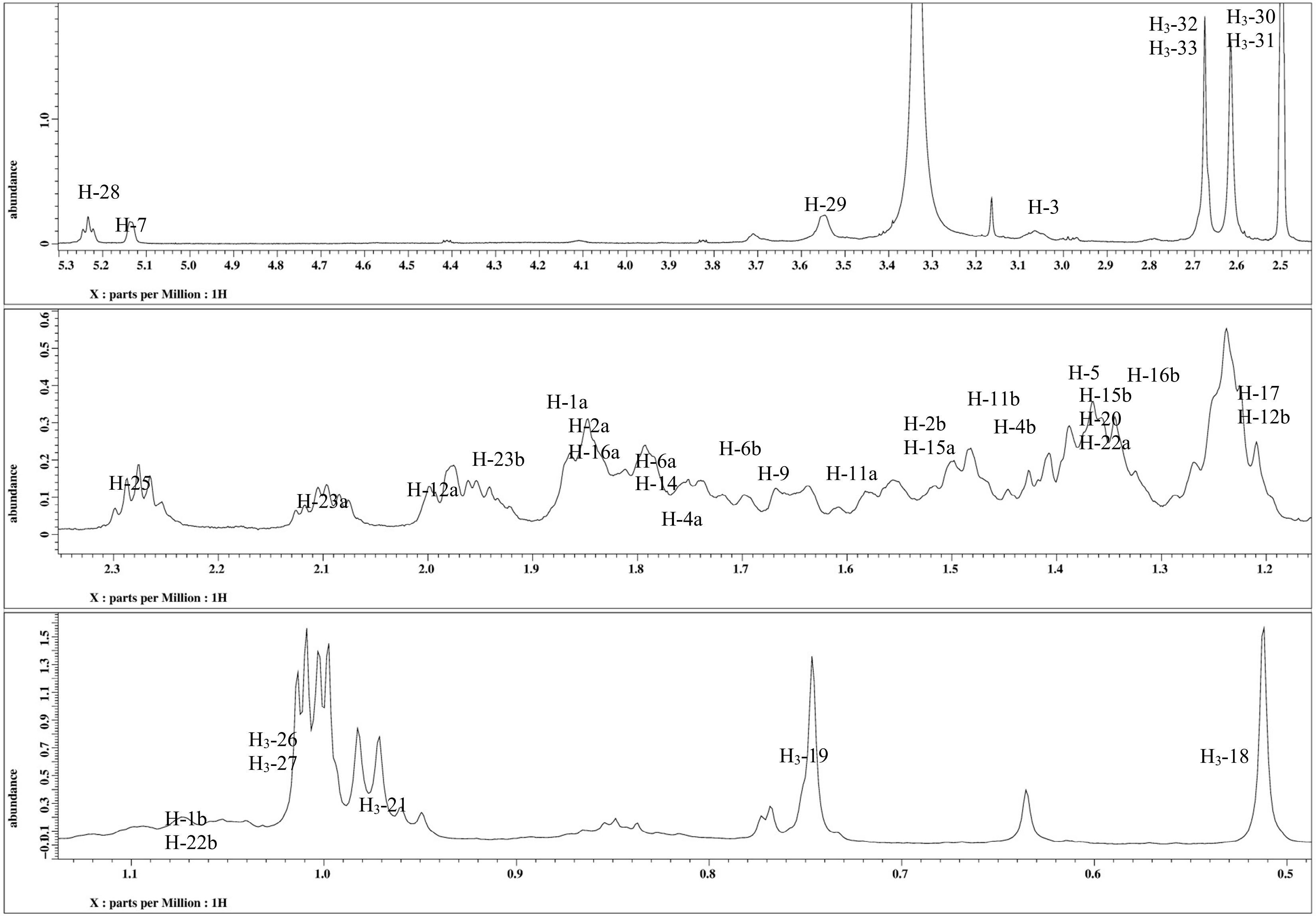

S17 ^{13}C NMR (150 MHz, DMSO-d_6) of Plakinamine P (**1**)

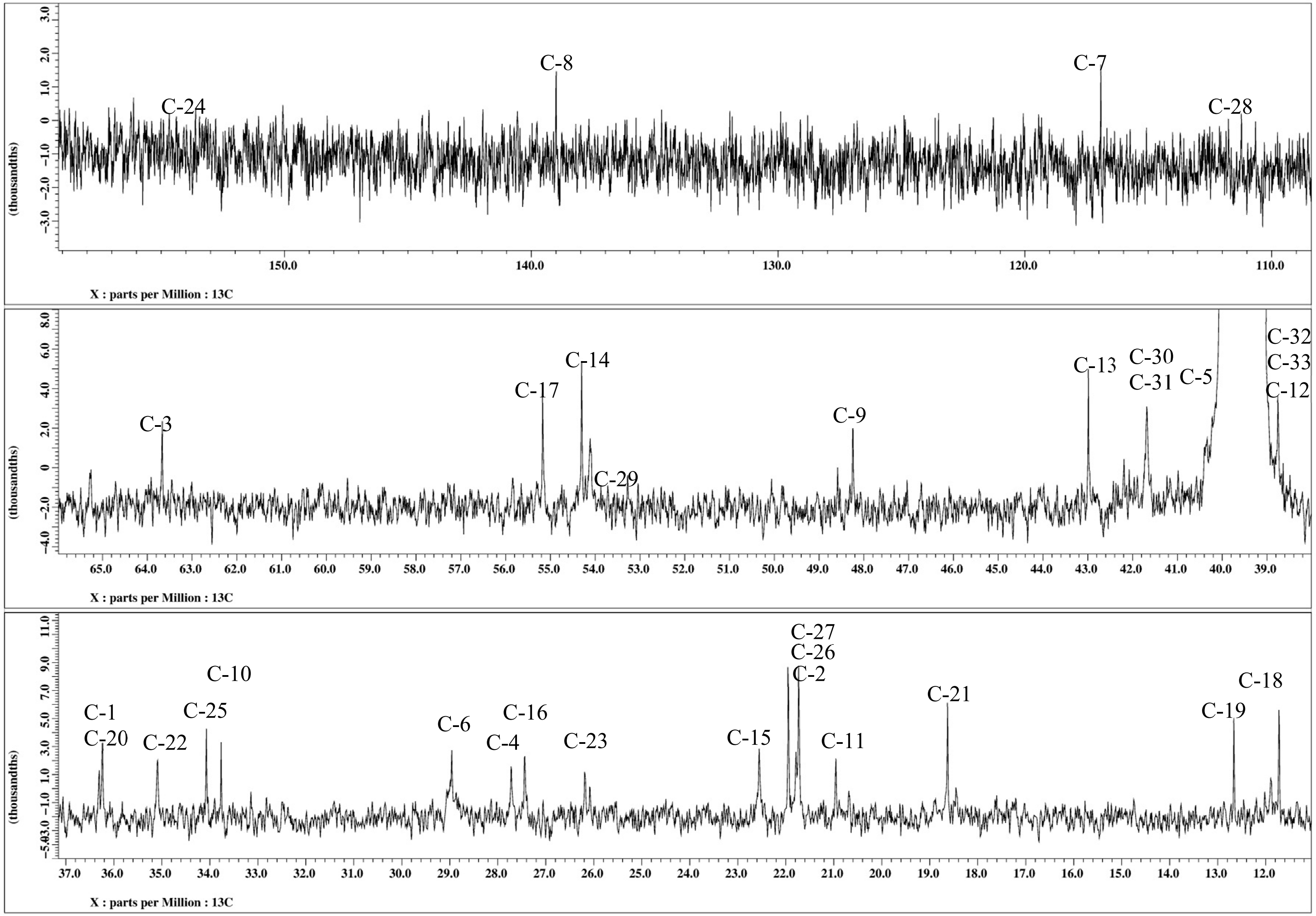

S18 gCOSY spectrum (600 MHz, DMSO-d_6) of Plakinamine P (**1**)

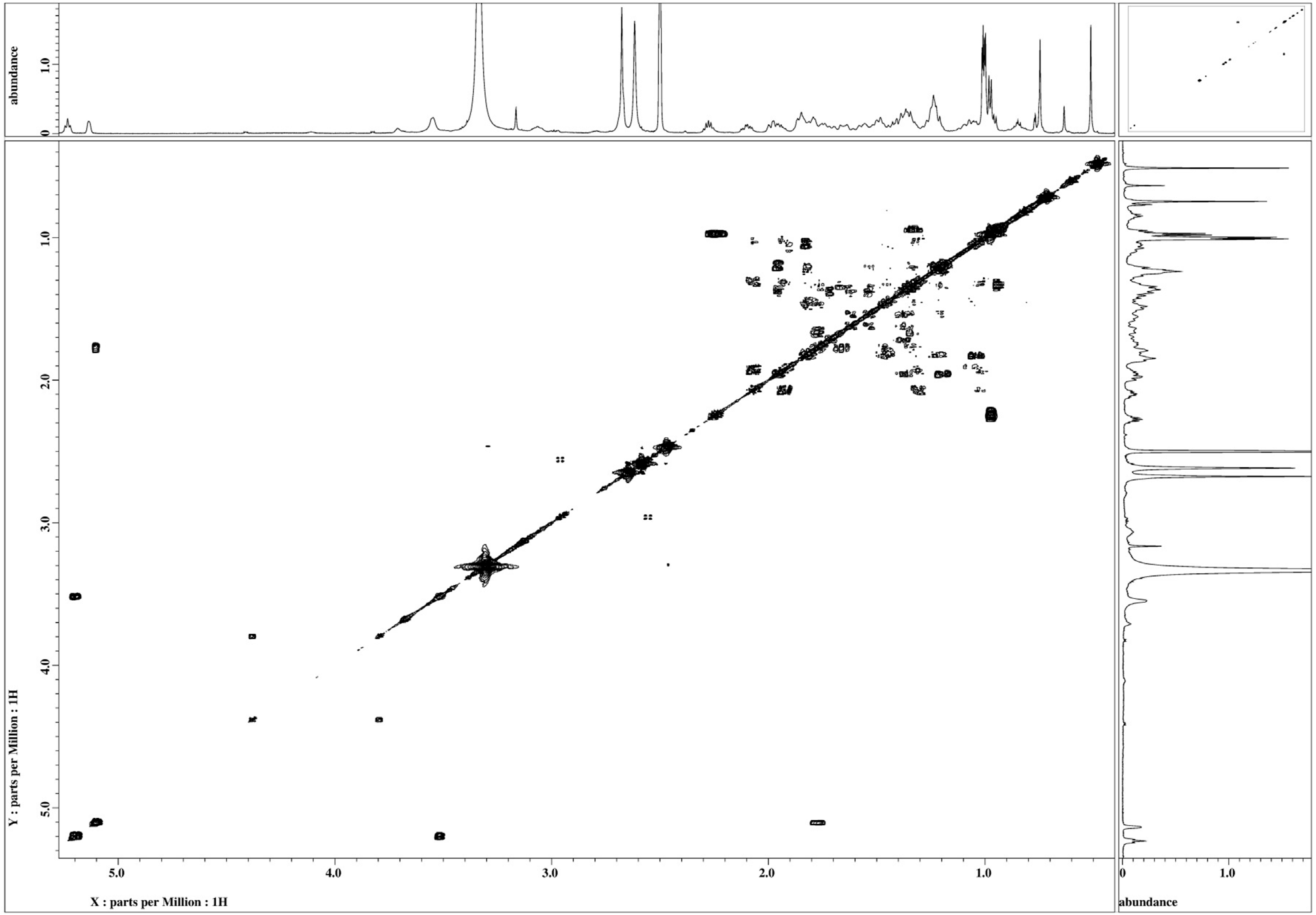

S19 edited gHSQC spectrum (600 MHz, DMSO-d_6) of Plakinamine P (**1**)

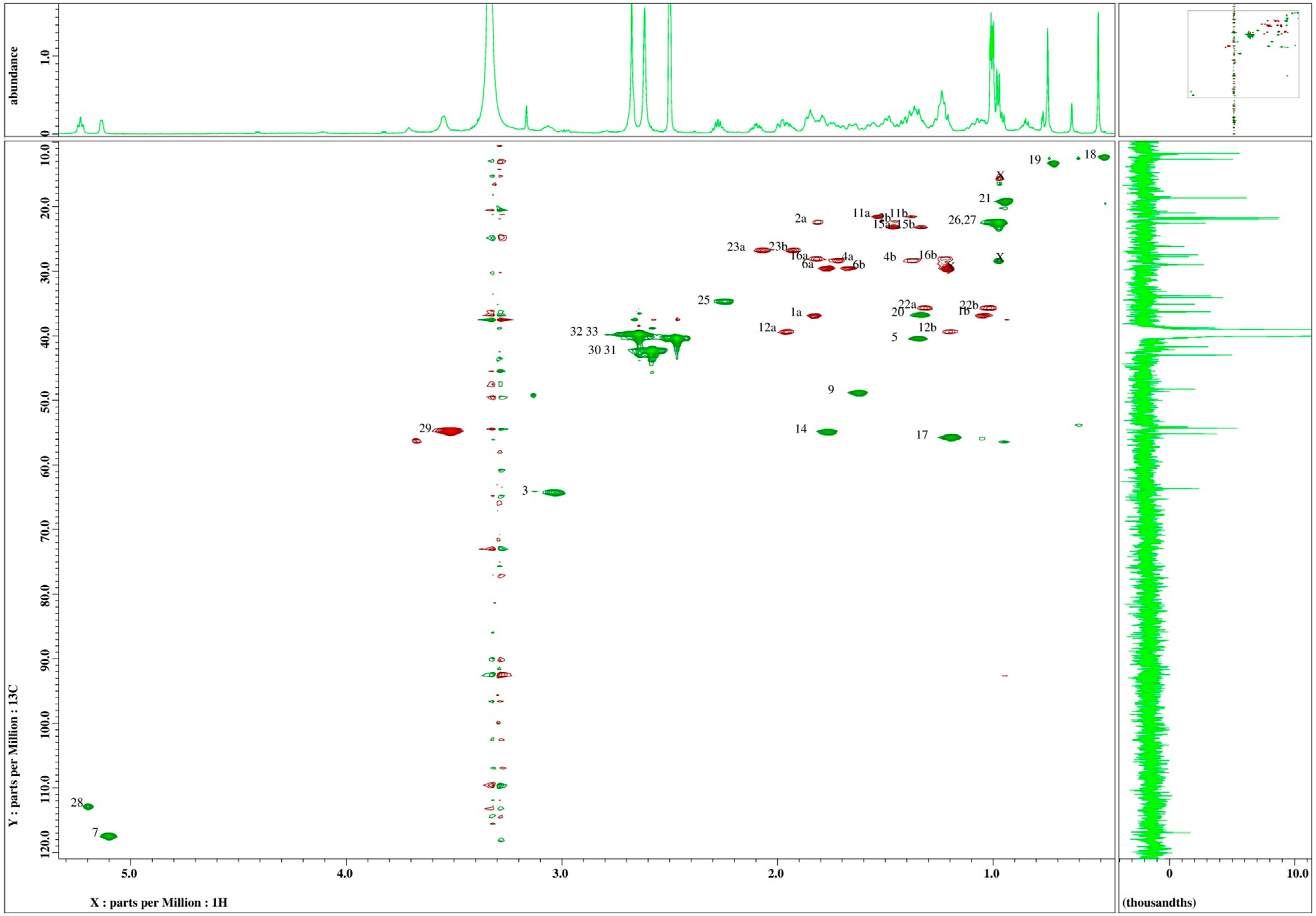

S20 gHMBC spectrum (600 MHz, DMSO-d_6) of Plakinamine P (**1**)

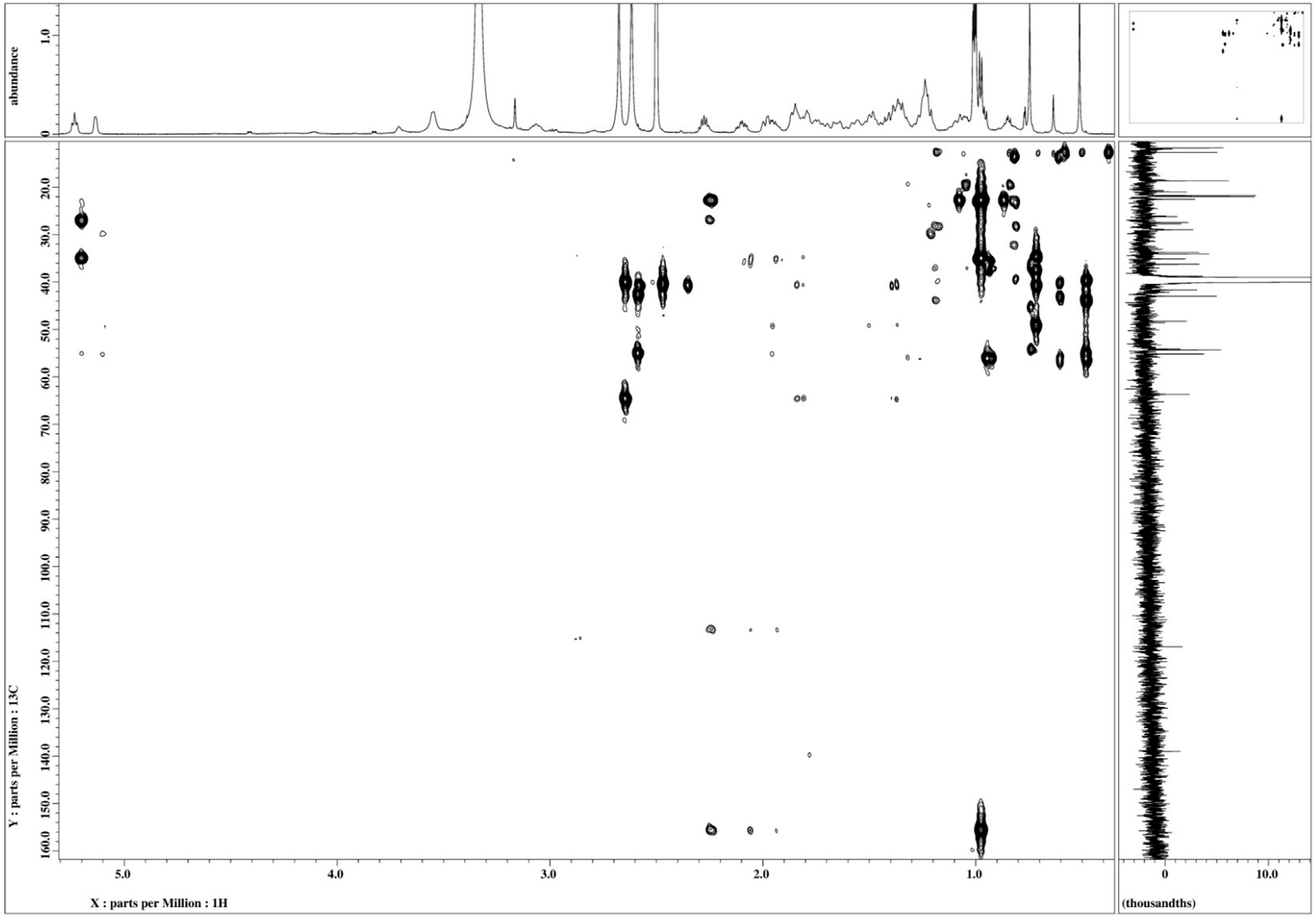

S21 NOESY spectrum (600 MHz, DMSO-d_6) of Plakinamine P (**1**)

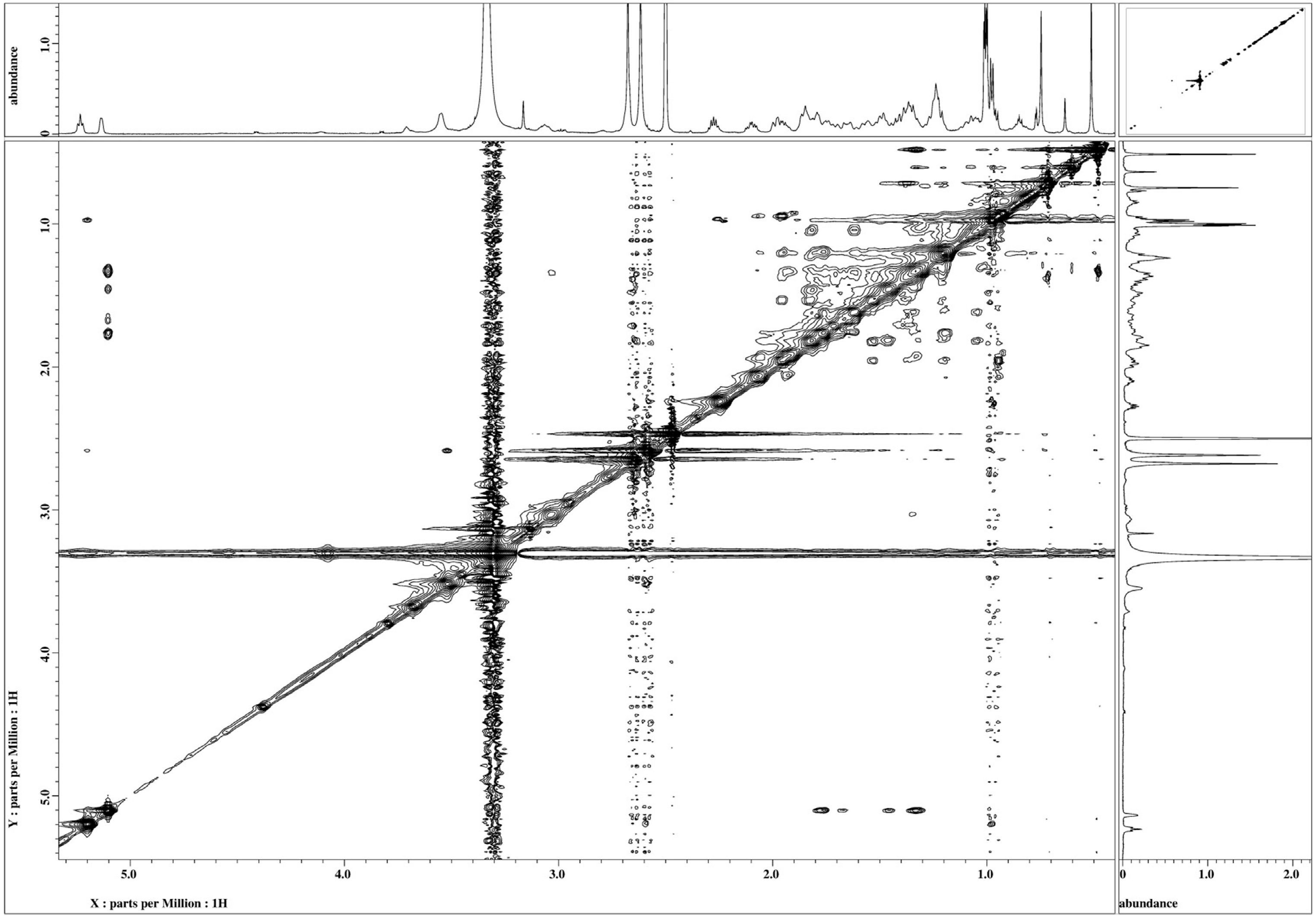

S22 Expansion of Plakinamine P (**1**) 2D-NOESY spectrum (600 MHz, DMSO-d_6) showing key correlations for H-3

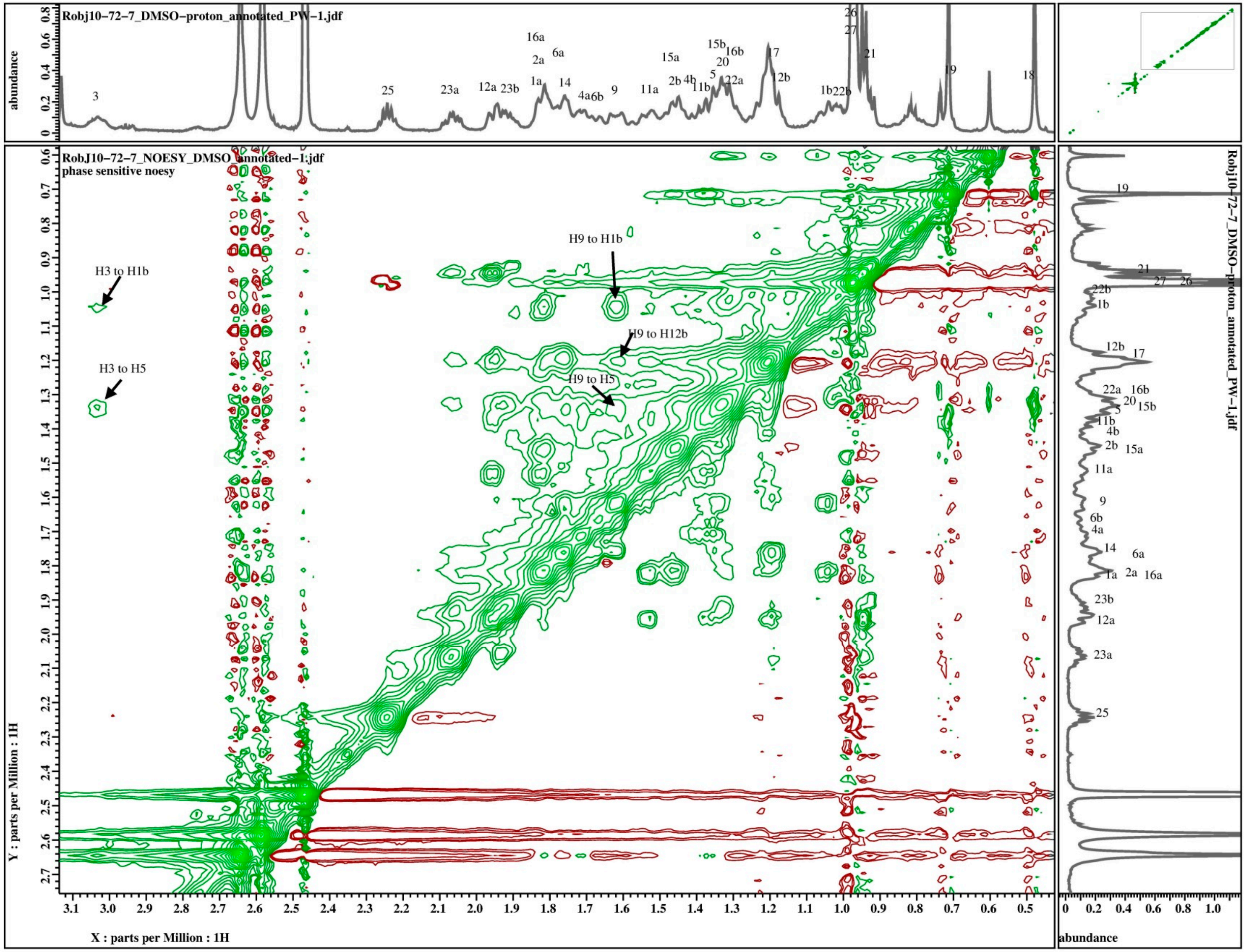

S23 ROESY spectrum (600 MHz, DMSO-d_6) of Plakinamine P (**1**)

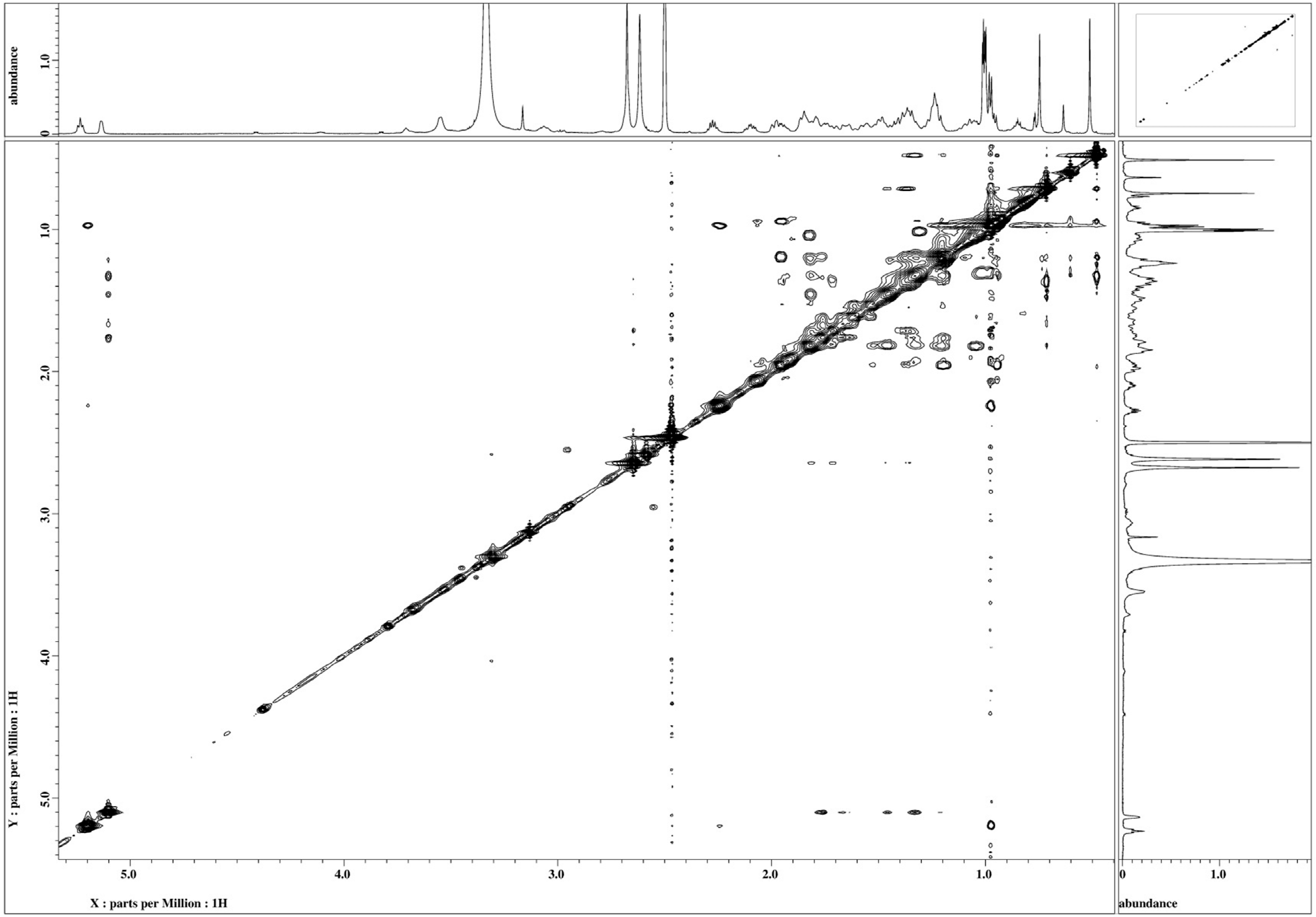

S24 Structure of plakinamine P (**1**) with chemical shifts in DMSO-*d*$_6$

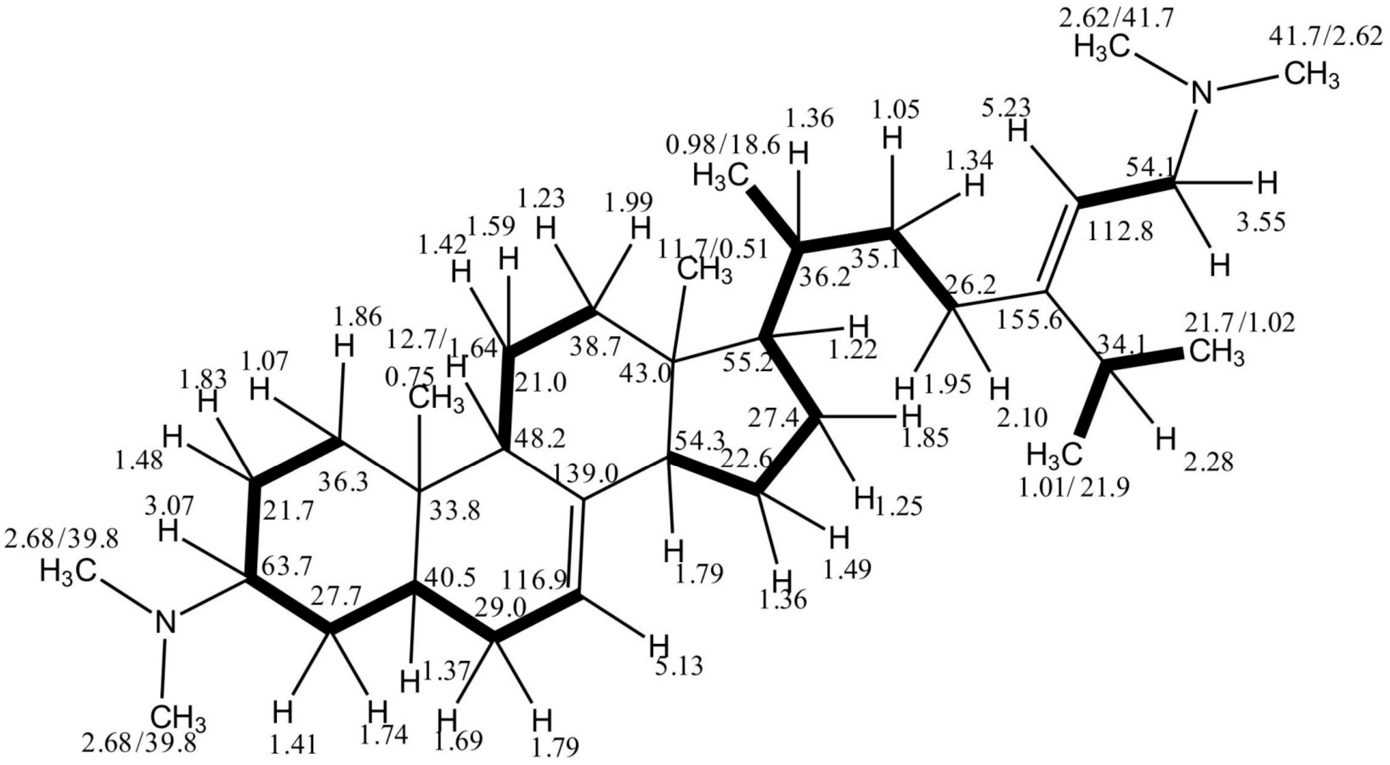

Table S2: Table of ^{1}H and ^{13}C NMR Data for plakinamine P (**1**) (600 MHz, DMSO-d_6)

position	δ_C, type	δ_H (J in Hz)	COSY	HMBC[a]	ROESY	NOESY
1a	36.3, CH_2	1.86, m	1b, 2b	3, 5	1b, 11a, 19	
1b		1.07, m	1a, 2ab		1a, 9	3, 9
2a	21.7, CH_2	1.83, m	1b		2b, 32/33	
2b		1.48, m	1ab, 2a		1a, 19, 32/33	
3	63.7, CH	3.07, br m	2b, 4ab		32/33	1b, 5
4a	27.7, CH_2	1.74, m	3, 4b		4b, 5, 32/33	
4b		1.41, m	3, 4a, 5		4a, 19	
5	40.5, CH	1.37, m	4ab, 6b		4a	3, 9
6a	29.0, CH_2	1.79, m	5, 6b, 7		5, 7	7
6b		1.69, m	5, 7		7	7
7	116.9, CH	5.13, br s	6ab	5, 6, 9, 14	6ab, 14, 15ab	6ab, 15ab
8	139.0, C					
9	48.2, CH	1.64, m	7, 11ab		1b, 12b, 14	1b, 5, 12b, 14
10	33.8, C					
11a	21.0, CH_2	1.59, m	9, 11b, 12ab	9	1a, 12a	12a
11b		1.42, m	9, 11a, 12ab		12a, 19	
12a	38.7, CH_2	1.99, m	11ab, 12b		11ab, 12b, 18, 21	11a, 12b, 21
12b		1.23, m	11ab		9, 12a, 14	9, 12a
13	43.0, C					
14	54.3, CH	1.79, m	15ab		5, 7, 12b	9
15a	22.6, CH_2	1.49, m	14, 15b, 16ab		7, 16a	7
15b		1.36, m			7, 16ab, 18	7
16a	27.4, CH_2	1.85, m			17	
16b		1.25, m			16a, 18	
17	55.2, CH	1.22, m	18			
18	11.7, CH_3	0.51, s	17	12, 13, 14, 17	11b, 12a, 15b, 16b, 17, 19, 20, 21	
19	12.7, CH_3	0.75, s	1b	1, 5, 9, 10	1a, 2b, 4b, 11b, 18[b]	
20	36.2, CH	1.36, m			18, 22b	
21	18.6, CH_3	0.98, d (6.9)	20	17, 20	12a, 18, 23a	12a
22a	35.1, CH_2	1.34, m	20, 22b, 23ab		21, 22b, 25	
22b		1.05, m	22a, 23ab		22a	
23a	26.2, CH_2	2.10, ddd (13.1, 13.1, 5.5)	22ab, 23b	24, 28	21, 23b, 26/27	21, 23b
23b		1.95, m	22ab, 23a	24, 28	22ab, 26/27	20, 23a
24	155.6, C					
25	34.1, CH	2.28, sep. (6.9)	26/27	23, 24, 26/27, 28	22a, 26/27, 28	26/27
26	21.9, CH_3	1.01, d (6.9)	25	24, 25, 27	23ab, 25, 28	

27	21.7, CH_3	1.02, d (6.9)	25	24, 25, 26	23ab, 25, 28	
28	112.8, CH	5.23, t (7.6)	29a	23, 25, 29	25, 26/27, 29, 30/31	25, 26/27, 30/31
29	54.1, CH_2	3.55, br s	28			30/31
30	41.7, CH_3	2.62, s		29, 31		
31	41.7, CH_3	2.62, s		29, 30		
32	39.8, CH_3	2.68, s		3, 33	1a, 2b, 4ab, 5	
33	39.8, CH_3	2.68, s		3, 32	1a, 2b, 4ab, 5	

[a]*g*HMBC correlations, optimized for 8 Hz, are from proton(s) stated to the carbons listed

[b]Though ^{1}H chemical shifts of 4b and 11b are very close, the ROESY correlations appear to go to both

S25 High resolution DART mass spectrometry data of plakinamine P (**1**)

HRMS of plakinamine P was measured on a JEOL AccuTOF-DART 4G using the DART attachment. The voltages for the atmospheric pressure interface are: Orifice 1 = 30 V; Orifice 2 = 5 V; Ring lens = 5 V; Ion guide voltage = 1000 V; Orifice 1 temperature = 100 °C. Mass spectra were stored at a rate of 1 spectrum per 0.4 seconds over the *m*/*z* range 100 to 1000 and a resolving power of >10,000 (FWHM). PEG 600 was measured by DART as a reference standard within the same data file as the DART measurements.

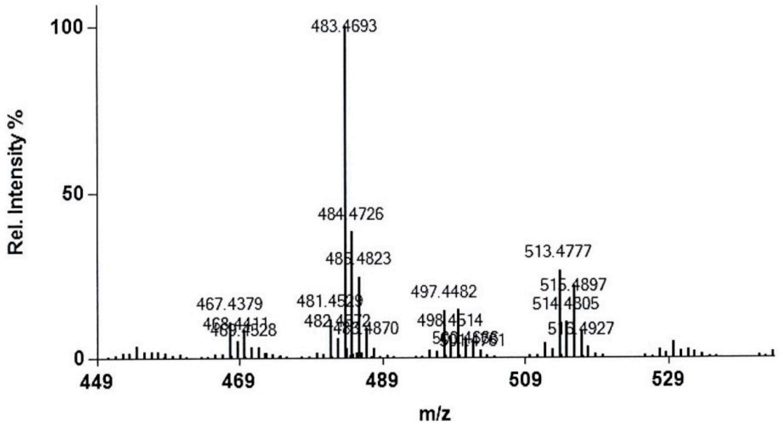

Mass Spectrum

Elemental Compositions
Element Limits: C 0/50 H 0/100 O 0/10 N 0/10
Tolerance: 5 mmuEven or odd electron ion or both: Even
Electron correction: None.Charges: 1
Minimum unsaturation: -1Maximum unsaturation: 100

Calc. m/z	Abund %	mmu	DBE	Composition
483.467825	100.000	-1.44	5.5	C33H59N2

S26 Picture and taxonomic description of the *Plakina* sp. used in the study

Plakina sp. Undescribed Caribbean species
Class Homoscleromorpha, Order Homosclerophorida, Family Plakinidae
Sample 25-V-93-3-9. HBOM Catalog Number 0004.001
The external morphology is bulbous to globular (1-2 cm thick, 2-6 cm in diameter), with a single oscule per bulb. Oscules are less than 4 mm wide with a tubular and darkened membrane projection. The surface of the sponge is smooth and the consistency is gelatinous and compressible. The specimen is light brown externally and tan in color internally, both in life and preserved. Spicules are in very low abundance and consist of small ramified calthrops (tetralophose and trilophose), rare smooth non-lophose calthrops, and small diod microrhabs (7-10 μm in length and <1 μm in width). Calthrops are regular in size, less than 20 μm in total size, with rays 8-10 μm x 2-3 μm. Trilophose calthrops usually have deformed rays with variation in the pattern of ramification between rays. The lophose calthrops are typical of the genus *Plakina*. There are five *Plakina* species currently recognized for the Caribbean: *Plakina elisa, Plakina nathaliae, Plakina tetralopha, Plakina jamaicensis and Plakina arletensis* The present *Plakina* specimen differs from those species in general morphology for which it has a much thicker growth, bulbous shape, and unique oscule morphology. The spicule combination, the tendency to deformed rays and diverse branching pattern within a spicule, and their low density in the body, are unique characteristics of this sample that indicate that it may be an undescribed *Plakina* species. Further study of the histology and genetics of this specimen will allow its distinction from the other Caribbean species.

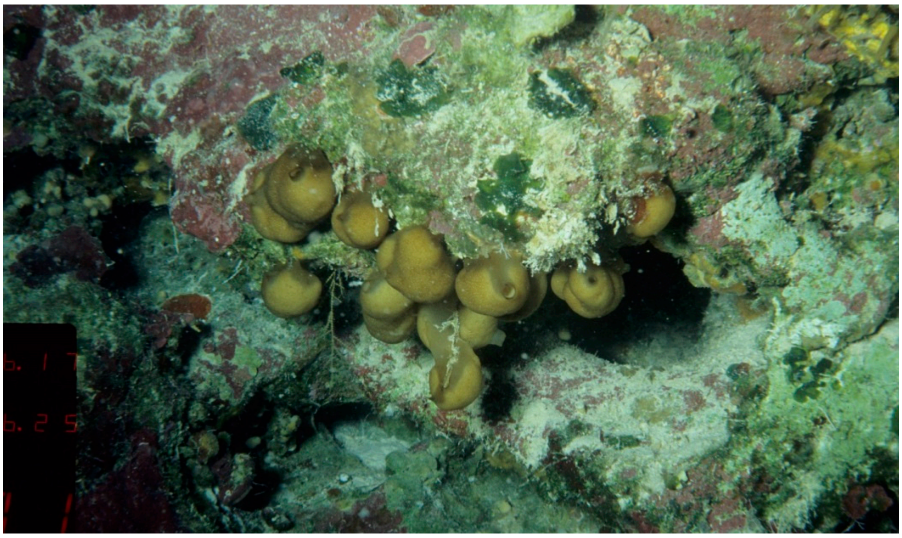

Figure S26A. Under water photograph of *Plakina* sp. collected from a cavern at 93 m deep, Crooked Island, Bahamas.

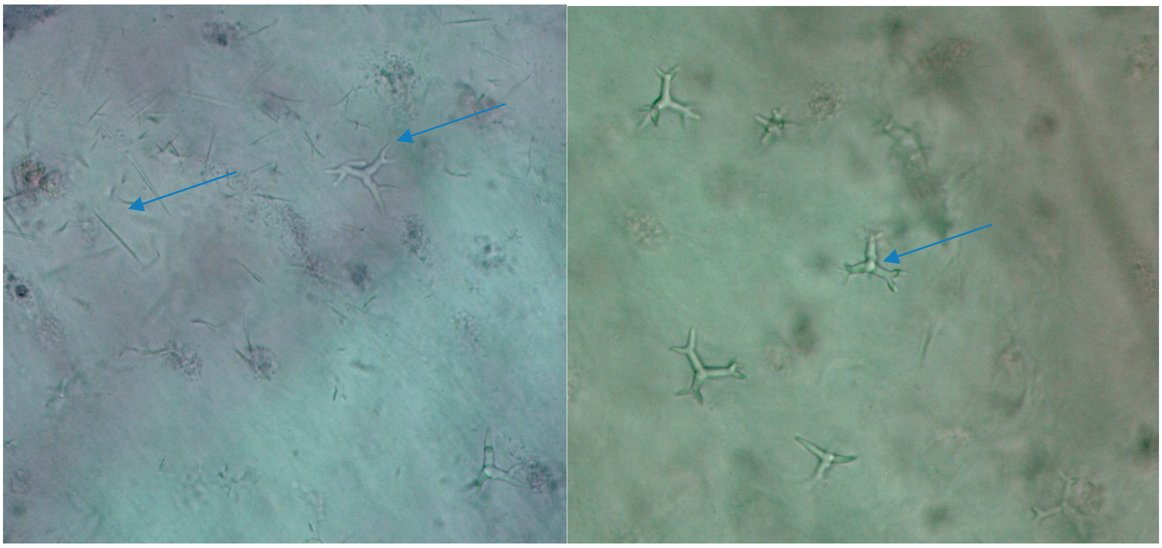

Figure S26B. Left image : trilophose calthrops with deformed rays, and diactineal microrhabds.
Right image tetralophose calthrops.

Ruiz, C., et al., *Descriptions of new sponge species and genus, including aspiculate Plakinidae, overturn the Homoscleromorpha classification. Zoological Journal of the Linnean Society*, 2017. **179**(4): p. 707-724
